# Gene signatures in wound tissue as evidenced by molecular profiling in the chick embryo model

**DOI:** 10.1186/1471-2164-11-495

**Published:** 2010-09-14

**Authors:** Fabienne Soulet, Witold W Kilarski, Philipp Antczak, John Herbert, Roy Bicknell, Francesco Falciani, Andreas Bikfalvi

**Affiliations:** 1 INSERM, U920, 3340 Talence, France; 2 Université Bordeaux I, 33 405 Talence, France; 3 Institute of Biomedical Research, University of Birmingham, Birmingham, B15 2TT, UK

## Abstract

**Background:**

Modern functional genomic approaches may help to better understand the molecular events involved in tissue morphogenesis and to identify molecular signatures and pathways. We have recently applied transcriptomic profiling to evidence molecular signatures in the development of the normal chicken chorioallantoic membrane (CAM) and in tumor engrafted on the CAM. We have now extended our studies by performing a transcriptome analysis in the "wound model" of the chicken CAM, which is another relevant model of tissue morphogenesis.

**Results:**

To induce granulation tissue (GT) formation, we performed wounding of the chicken CAM and compared gene expression to normal CAM at the same stage of development. Matched control samples from the same individual were used. We observed a total of 282 genes up-regulated and 44 genes down-regulated assuming a false-discovery rate at 5% and a fold change > 2. Furthermore, bioinformatics analysis lead to the identification of several categories that are associated to organismal injury, tissue morphology, cellular movement, inflammatory disease, development and immune system. Endothelial cell data filtering leads to the identification of several new genes with an endothelial cell signature.

**Conclusions:**

The chick chorioallantoic wound model allows the identification of gene signatures and pathways involved in GT formation and neoangiogenesis. This may constitute a fertile ground for further studies.

## Background

Different physiological as well as pathological conditions trigger tissue remodeling including surgery, infection, chemical or physical burns, ischemia or immunological reaction [1]. The restoration of tissue integrity involves alteration in tissue elasticity, interstitial fluid pressure and oxygen tension, which is normalized by vascularization of the affected region [2]. Revascularization is accomplished by the ingrown of the granulation tissue (GT) that is composed of a dense network of enlarged vessels forming specific and leaky temporary vasculature [3]. When not disturbed, GT vasculature is normalized during course of scarification. The healing process proceeds according to that general pattern e.g. in the skin but also during regenerative healing after brain or myocardium stroke [4]. Wound healing can be perturbed by pathological changes that include ulceration, hypertrophic scaring or keloids formation and fibrosis [5]. Modern therapy requires the targeting of drugs directly to the site of interest and to accomplish that goal in systemic treatment, the molecular signatures distinguishing the expanding vasculature of the GT from the normal vessels need to be known.

The chicken embryo model has been widely used in developmental biology to understand vascular development and to test the effect of molecules predicted to interfere with the angiogenic process or lymphangiogenesis [6]. For example, the effect of flow on vessel ontology such as venous or arterial patterning has been elucidated using the chicken chorioallantoic membrane (CAM) [7]. Furthermore, the effect of different angiogenesis stimulators such as VEGF-A, VEGF-C or inhibitors has been tested in the chick embryo. Adult wound healing involves movement from the epidermis and connective tissue and the recruitment of inflammatory and immunocompetent cells. Embryonic wound healing also involves wound contraction, followed by re-epithelialization but without recruitment of immunocompetent cells. The inflammatory response in wound healing is crucial for fighting infection so that tissue damage does not lead to death through septicaemia. But, aside from this role, recruitment of leukocytes may more negatively impact wound healing. Indeed, knockout and knockdown studies suggest that immun cells do not promote wound healing and their depletion can even enhance it [1,8]. Thus, models of embryonic wound healing will evidence gene regulations that are crucial for the healing process and independent from the perturbation induced by immunocompetent cells. However, neutrophile-like inflammatory cells and monocyte-like cells are accumulated in growth factor-stimulated CAM which may participate in the wound healing process[9].

Kilarski et al. [10] have developed a method to investigate GT formation in the CAM. This model has allowed a better understanding of the formation of the vasculature during GT formation [10]. The major advantages of CAM wound healing model is that the CAM is composed of blood vessels and enclosed within 2 layers of epithelium and a fibroblast matrix. This is in contrast to a skin model, for example, where there are multiple cell and tissue types (epidermis, dermis and subdermis). Wound healing in the CAM model reflects primary changes in vasculature and in stromal fibroblasts that is not affected by "noise" from other cell types. Furthermore, a matched control can be obtained from the same CAM.

Modern functional genomics approaches may facilitate a better understanding of the molecular events involved in tissue morphogenesis and allow the identification of molecular signatures and pathways. We have recently applied transcriptomic profiling to elucidate the molecular signatures involved in the development of the normal chicken chorioallantoic membrane and in tumors engrafted onto the CAM [11,12]. Furthermore, engraftment of human tumour tissue onto the CAM, followed by transcriptomic analyses with both human and chicken microarrays, enables the gene signatures of both the host stroma and the human tumour to be distinguished. We have now extended our earlier studies by performing a transcriptome analysis in the "wound model" of the chicken CAM. This has allowed us to identify gene signatures involved in GT formation and neoangiogenesis. These results further indicate that the chicken embryo model is an excellent tool for discovering networks that are associated with granulation-tissue formation and tissue repair.

## Results and Discussion

### Wound induction in the chicken CAM

**Figure 1:**
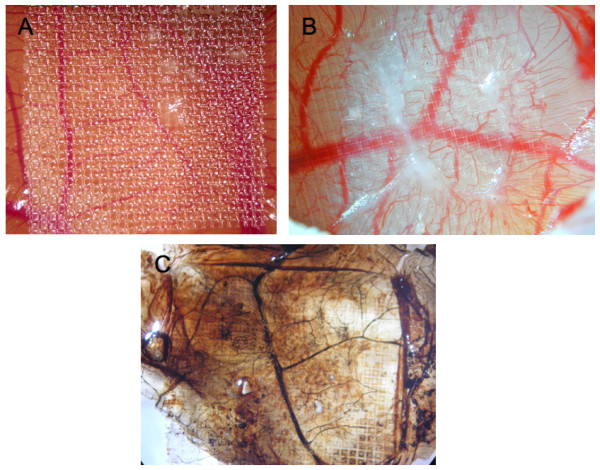
**Wound Model of the chicken chorioallantoic membrane**. Panels A and B of this figure display granulation tissue 6 days after injury (B is a larger magnification of A). In panel C, also showing granulation tissue 6 days after injury, Indian ink was injected to visualize perfused blood vessels (vessels are now black).

Chick embryos were cultured for 10 days and CAMs were inflicted by parallel scalpel superficial cuts of 1 cm area, followed with a subsequent scarping off of the epithelium of the injured chorioallantoic membrane. The wound area was then covered with 1.5 cm square nylon grid (figure 1A). As seen in figure 1B, significant GT was formed and grew through the nylon grid in response to injury. This GT contained a significant amount of blood vessels with abnormal morphology (tortuous blood vessels). Newly formed blood vessels within the CAM became functional since injection of Indian ink clearly evidenced newly perfused blood vessels in the GT (figure 1C).

**Figure 2:**
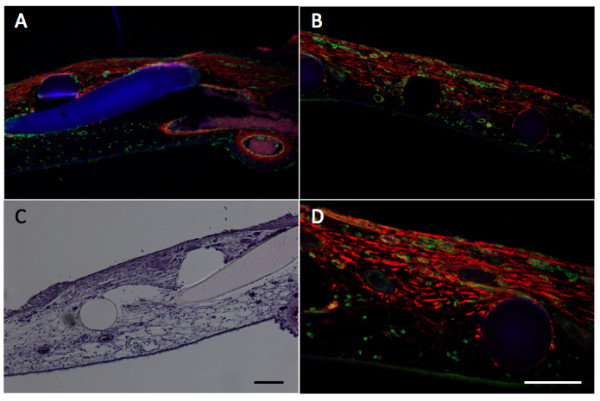
**Histology and immunohistology in the wound model**. Wounding is performed as indicated in Methods and analyzed by histology and immunohistology. Panel A and B, immunohistological analysis of α2-smooth muscle actin (anti-α2-SM antibody) in red, vessels are stained in green (SNA-isolectin) and nuclei in blue (Dapi) of two different areas of the wounded CAM (10× magnification). Panel C, Hematoxylin-eosin staining (10× magnification. Panel D is a higher magnification (20× magnification) of B. Scale bars, 100 μm. The figure clearly shows the infiltration of a2-SM positive myofibroblasts and of blood vessels in the wounded area. The grid is visible in blue (A), or as black (B,D) and white holes (C) or in rosa (C).

The CAM wound model has been established to analyze GT formation and the role of invading fibroblasts and blood vessels in this process [10]. It has been found that tissue tension generated by activated fibroblasts or myofibroblasts during wound contraction, mediated and directed translocation of the vasculature. This vasculature can be expanded secondarily by elongation and vessel enlargement, and finally, through splitting and sprouting. To verify the presence of myofibroblasts in our experimental set up, we performed immunohistology using anti-α2-somooth muscle actin antibodies. The invasion of α2-smooth muscle (α2-SM) actin positive myofibroblasts and of blood vessels in the wounded area was clearly visible and is depicted in figure 2.

Other chick wound models that have been proposed such as epithelial regeneration models at the surface of the embryo such as at the wing bud or the midbrain region [8,13,14]. These models have helped to characterize some of the morphological and molecular events occurring during embryonic tissue repair involving actin cable assembly and the Rho kinases[8,14]. Tissue wound contraction is present in this model to some extent, however without the presence of α2-SM positive myofibroblasts[8]. Another model is characterized by the removal of only the peridermal layer. Wound closure in this model is essentially driven by the conversion of the basal layer, from monolayer to multilayer [15].

The advantage of our model is that it clearly distinguishes between preexisting and newly formed tissue and vasculature and that an ingrowth of α2-SM actin positive myofibroblasts is observed. Furthermore, recruitment and translocation of the vasculature in the wound area can be clearly envisioned. This allows us to perform transcriptomic analysis after wounding to establish which genes are important players in this process.

### Gene signatures in wound tissue by molecular profiling

**Figure 3:**
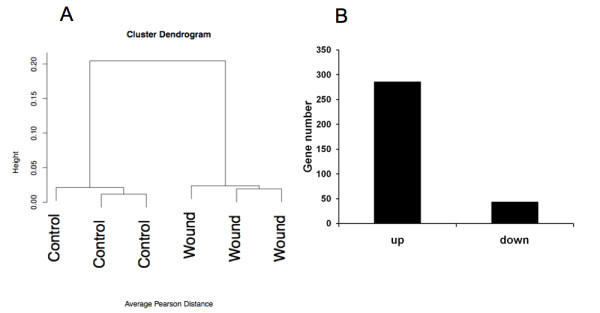
**Global analysis of the transcriptomic data**. Panel A displays hierarchical clustering of the wounds versus the control microarray results. In panel B, the quantitative representation of up and down-regulated genes in granulation tissue in comparison with control tissue is displayed.

We therefore extracted RNA from eggs 6 days post-wounding. We also extracted RNA from areas where no injury was inflicted to the CAM from the same eggs. Thus, each wound had a matched control from the same egg and three eggs were used for hybridization on each chip with their own controls (6 samples in total). Data were normalized using the robust multi-averaging method, part of the Affymetrix library in the statistical programming language R. Hierarchical clustering, performed on the three wounded and non-wounded samples, indicated separate clustering of wounded and non-wound CAM tissue (figure 3A). This indicates good quality of the samples collected and of hybridizations. We observed a total of 282 genes up-regulated and 44 genes down-regulated, applying a FDR < 5% and a fold change > 2 (figure 3B).

Up-regulation as high as 79 fold was observed. Among the most up-regulated genes, we observed: fatty acid binding protein 4 FABP4 (79 fold), retinol binding protein 7 (RBP7) (74 fold), transthyretin (TTR) (37fold), osteopontin (SPP1) (30 fold), neutrophile cytosolic factor (NCF2) (25 fold), chemokine ah221 (LOC417536) (10 fold) and cysteine-rich secretory protein (CRISP3 (8 fold)) (Table 1). Among the most down-regulated genes, we observed: inter-alpha (globulin) inhibitor H5 (ITIH5) (0.11 fold), Collagen type VIII a1 (0.15 fold), testican (SPOCK1) (0.29 fold), Atonal homolog 8 (ATOH8) (0.29 fold), laminin a1 (LAMA1) (0.32 fold), CXCL12 (0.32 fold), C1q and TNF-related protein 1 (C1QTNF1) (0.32 fold) and plexin A2 (PLXNA2) (0.33 fold) (Table 2).

**Table 1:** List of 20 most induced genes in granulation tissue

Affymetrix ID	UniGene ID	Gene Name	Gene Symbol	Fold Change
Gga.4939.1.S1_s_at	Gga.4939	fatty acid binding protein 4, adipocyte	FABP4	79.23
Gga.9386.1.S1_at	Gga.9386	retinol binding protein 7, cellular	RBP7	73.82
Gga.17686.1.S1_at	Gga.41504	keratin 75	KRT75	59.38
Gga.2620.1.S1_at	Gga.2620	transthyretin	TTR	36.7
Gga.3551.1.S1_at	Gga.3551	secreted phosphoprotein 1 (osteopontin, bone sialoprotein I, early T-lymphocyte activation 1)	SPP1	30.01
Gga.17647.1.S1_at	Gga.34552	neutrophil cytosolic factor 2 (65 kDa, chronic granulomatous disease, autosomal 2)	NCF2	25.09
Gga.7228.1.S1_at	Gga.7228	carboxymethylenebutenolidase homolog (Pseudomonas)	CMBL	14.33
Gga.11640.1.S1_at	Gga.11640	succinate receptor 1	SUCNR1	11.04
GgaAffx.10393.1.S1_at	Gga.46851	cytochrome b-245, beta polypeptide (chronic granulomatous disease)	CYBB	10.25
Gga.9133.1.S1_at	Gga.9133	Chemokine ah221	LOC417536	9.95
Gga.6239.1.S1_at	Gga.6239	regulator of G-protein signalling 1	RGS1	9.28
Gga.11456.1.S1_at	Gga.11456	cystatin A (stefin A)	CSTA	9.25
Gga.3383.1.S1_at	Gga.3383	lipopolysaccharide-induced TNF factor	LITAF	9.16
Gga.729.1.S1_at	Gga.729	mature avidin	LOC396260	8.41
Gga.1158.3.S1_a_at	Gga.1158	HOP homeobox	HOPX	8.41
GgaAffx.6592.1.S1_at	---	---	---	8.36
GgaAffx.25031.1.S1_at	Gga.19498	cysteine-rich secretory protein 3	CRISP3	8.29
Gga.17679.1.S1_s_at	Gga.39008	similar to immunoglobulin-like receptor CHIR-AB3 -B4 -B5 -B	LOC425449	8.2
GgaAffx.11785.1.S1_s_at	Gga.9879	lipase A, lysosomal acid, cholesterol esterase (Wolman disease)	LIPA	8.17
Gga.5743.1.S1_at	Gga.5743	lymphocyte antigen 96	LY96	8.09

**Table 2:** List of 20 most down-regulated genes in granulation tissue

Affymetrix ID	UniGene ID	Gene.Name	Gene Symbol	Fold Change
Gga.9732.1.S1_at	Gga.43260	inter-alpha (globulin) inhibitor H5	ITIH5	-9.09
Gga.3013.1.S1_at	Gga.3013	collagen, type VIII, alpha 1	COL8A1	-6.67
Gga.3652.1.S1_at	Gga.3652	C-type lectin domain family 3, member B	CLEC3B	-4.35
GgaAffx.8087.2.S1_s_at	---	---	---	-4.17
Gga.15966.1.S1_at	Gga.35123	ankyrin 2, neuronal	ANK2	-3.85
Gga.9166.1.S1_at	Gga.9166	Finished cDNA, clone ChEST252j10	---	-3.57
Gga.16835.1.S1_at	Gga.15599	sparc/osteonectin, cwcv and kazal-like domains proteoglycan (testican) 1	SPOCK1	-3.45
Gga.8360.1.S1_at	Gga.36205	Atonal homolog 8 (Drosophila)	ATOH8	-3.45
GgaAffx.26547.1.S1_at	---	---	---	-3.33
Gga.170.1.S1_at	Gga.170	wingless-type MMTV integration site family, member 2B	WNT2B	-3.33
Gga.6141.1.S1_at	Gga.6141	immunoglobin superfamily, member 21	IGSF21	-3.33
Gga.12209.1.S1_at	Gga.12209	Chromosome 4 open reading frame 31	C4orf31	-3.12
GgaAffx.24366.1.S1_at	Gga.23904	laminin, alpha 1	LAMA1	-3.12
Gga.9513.1.S2_at	Gga.9513	Chemokine (C-X-C motif) ligand 12 (stromal cell-derived factor 1)	CXCL12	-3.12
GgaAffx.7505.1.S1_at	Gga.29269	C1q and tumor necrosis factor related protein 1	C1QTNF1	-3.12
Gga.19875.1.S1_at	Gga.23851	plexin A2	PLXNA2	-3.03
Gga.8241.1.S1_at	Gga.8241	transcription factor 21	TCF21	-2.86
Gga.9024.1.S1_at	Gga.43042	chromosome 8 open reading frame 22	C8orf22	-2.86
Gga.618.1.S1_at	Gga.43121	cytochrome P450 1A4	CYP1A4	-2.78
GgaAffx.3294.1.S1_at	Gga.37857	similar to SYT9 protein	LOC423026	-2.7

**Figure 4:**
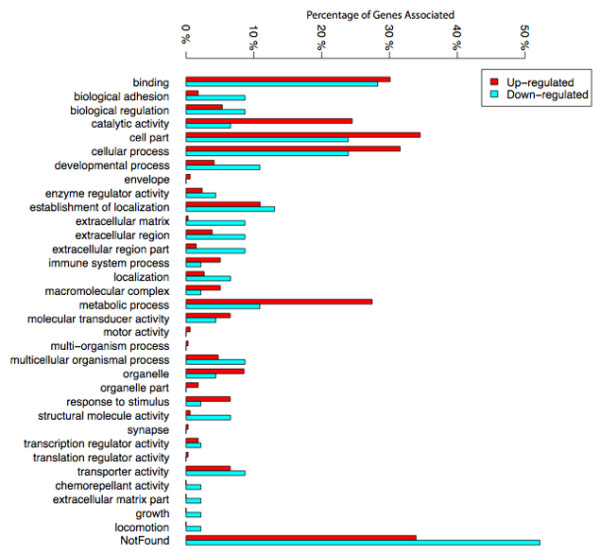
**Gene ontology**. This figure portrays the gene ontology of up and down-regulated genes in granulation tissue in comparison with control tissue. Numbers represent the percentages of genes identified associated with a biological process, a cellular component or a molecular function.

In order to analyze potential functional trends in the gene lists identified, we performed a functional analysis of the lists of differentially expressed genes. With the purpose to describe the overall representation of functions differentially modulated in response to wounding, we first computed the number of genes represented in the most common gene ontology (GO) categories. As seen in figure 4, the most abundant categories found in this analysis were "binding", "catalytic activity", "cell part", "cellular processes" and "metabolic processes". There was a slight preponderance of up-regulated genes within the first three categories. However, within "metabolic processes" the majority of genes were up-regulated rather than down-regulated.

Furthermore, differences in four other categories related to extracellular matrix and adhesion ("biological adhesion", "extracellular matrix", "extracellular region" and "extracellular region part") were quite striking. In these categories, down-regulated genes were much more abundant. In addition to this analysis, and in order to identify functional categories enriched in our gene lists, we also performed a functional analysis using the web based tool, DAVID [16,17]. This procedure clusters genes with similar functions (defined by similar Gene ontology or KEGG pathway terms) into annotation clusters (Additional file 1). It then assesses whether these "functional clusters" are over-represented in the list of genes differentially regulated. We submitted the up and down regulated genes separately and summarized the results in table 3. Due to the relatively low number of significant genes at 5% FDR level, we have also verified that results of the analysis were similar at less stringent FDR thresholds (10% and 15%FDR). Generally no new functions emerged when the FDR was increased but as expected, each function increased in significance and enrichment.

**Table 3:** DAVID analysis summary

UP		LO	
Enrichment Score	Function	Enrichment Score	Function
4.64	antigen processing and presentation pf petide or polysaccharide antigen via MCH class II	2.55	Extracellular matrix
2.43	phosphoinositide binding	1.06	Developmental process
2.28	transmembrane		
1.9	Cytokine/chemokine activity		

This table shows a summary of the DAVID analysis. The functional terms most representing the annotation cluster is shown in columns 2 and 4 with the respective enrichment scores in columns 1 and 3. The higher the enrichment score, the more interesting the enriched function.

The expression of a number of cytokines or chemokines was up-regulated after wounding. Some of them are represented in the GO term "Cytokines-Chemokines". Among the others clearly associated with this category are IL10R, IL4R, chemokine ligand 20 (CCL20), CX3CR1 and IL1β. Components of the major histocompatibility complex (MHC) were also found up-regulated after wounding. The latter may represent antigen presentation as a consequence to injury. This transcriptional response is not likely to be part of the immune system but may be associated to other cellular types of the GT such as endothelial cells or fibroblasts. Interestingly, the expression of some components of the extracellular matrix and genes involved in developmental processes were decreased. These include Wingless-type MMTV integration site family member 2B (WNT2B), fibrillin-1(FBN1), laminin-α1 (LAMA1), collagen VIII (COL8A1), FRAS1-related extracellular matrix-1 (FREM1), Cysteine-rich transmembrane BMP regulator 1 (CRIM1), Semaphorin 3G (SEMA3G) or Eph receptor A7 (EphA7). This indicates that synthesis of these components is no more required when significant GT formation has occurred. For the list of individual genes represented in our gene lists and belonging to these enriched categories, see additional files 2 and 3.

### Network analysis of genes modulated in response to wounding

In order to identify the structure of regulatory networks underlying response to wounding we performed Ingenuity Pathway Analysis (IPA). IPA identifies gene interaction networks representing potential regulatory pathways by integrating lists of differentially expressed genes with a vast public domain literature database, representing several types of gene-gene interactions. Contrary to gene ontology analysis IPA networks represented gene interactions linked to specific mechanisms (e.g. transcriptional activation, protein-protein interactions, etc).

Additional file 4 summarizes the results of this analysis by listing the most significant networks identified. The Ingenuity category with the best scores (> 20) were "Cardiovascular disease, organismal injury, tissue morphology" (score: 50), "Free Radical Scavenging, Cellular Movement, Hematological System Development and Function» (score: 45), « Inflammatory Disease, Respiratory Disease, Carbohydrate Metabolism » (score: 37), « Cellular Development, Hematological System Development and Function, Immune and Lymphatic System Development and Function » (score: 31), « Lipid Metabolism, Small Molecule Biochemistry, Vitamin and Mineral Metabolism » (score: 24), « Cancer, Immune and Lymphatic System Development and Function, Gene Expression » (score: 22).

**Figure 5:**
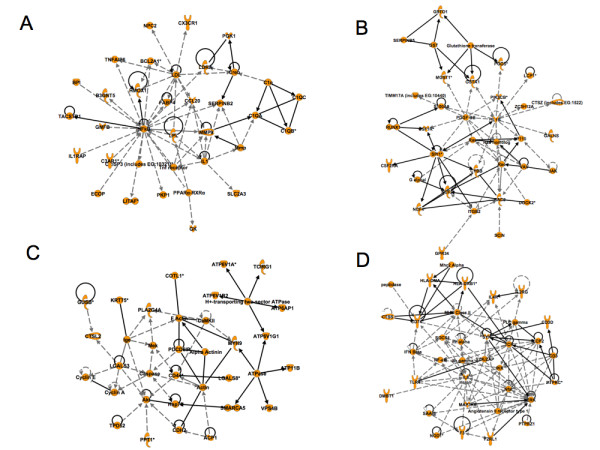
**A-D: Representation of 4 networks (Networks 1, 2, 3 and 4) derived from the Ingenuity Pathway Analysis (IPA)**. This figure displays an analysis, which depicts the central role of cytokines in granulation tissue and wound healing. The network categories are indicated in each figure. A, Network 1 (up-regulated genes) Cardiovascular Disease, Organismal Injury and Abnormalities, Tissue Morphology; B, Network 2 (up-regulated genes): Free Radical Scavenging, Cellular Movement, Hematological System Development and Function; C, Network 3 (up-regulated genes): Inflammatory Disease, Respiratory Disease, Carbohydrate Metabolism; D, Network 4 (up-regulated genes): Cellular Development, Hematological System Development and Function, Immune and Lymphatic System Development and Function.

The most significant networks identified with the best IPA scores are Network 1, 2, 3 and 4 (figures 5A-D). Network 1 and 2 rank top in the IPA score and reflect gene regulations involved in organismal injury, tissue morphology and cellular movement such as IL1, IL1b, ITGB2, PDGFBB, Rac, Ras, MMP9, PPAR, SERPINB2&B5, IL1RAP or CXC3CR1. Network 3 depicts several genes that are involved in cell cycle regulation (cyclines A, E), cell movement (CD44, caspase). Network 4 involves regulations implicated in cellular development and cell-cell interactions (IL8, IL2R, IFN-γ etc). The represented networks are associated with the production and response of cytokine components. More specifically, network 1 and 2 show the importance of IL-1 in the response to wounding. Our results suggest that role for this cytokine in the development of tissue repair and possibly in the onset of angiogenesis. It is known that IL-1β interacts with endothelial cells and induces VEGF and iNOS expression [18]. IL-1β also signals through IRAKs in vascular endothelial cells and induces genes in the VEGF pathway [19,20]. Furthermore, IL-1β induces fibrosis after radiation in experimental models, which indicate a possible interaction of IL-1β with fibroblasts[21]. In addition, IL1β concentrations are elevated in chronic wounds in patients [22]. Thus, IL-1β may regulate fibroblast proliferation and migration during GT formation. Among the other central cytokines are IL-8 and interferon-γ (network 4 and 6, see also additional file 5). The interaction of these cytokines with the vasculature and the stroma is well established [23,24]. Furthermore, IL-8 expression is elevated in chronic wounds but not acute wounds, which supports a role of this cytokine in GT formation [22]. IL-8 is a powerful angiogenic chemokine implicated in vessel formation in cancer, tissue repair and inflammation. It stimulates VEGF expression and autocrine activation of VEGFR2 in vascular endothelial cells [25]. Interferon-γ may interact with stroma fibroblasts and paradoxically negatively impact on angiogenesis [26]. It has been described that the induction of the CXC chemokine interferon-γ-inducible protein-10 (IP10) regulates the reparative response following myocardial infarction [27]. Interferon-γ may, thus, modify the cellular composition of the healing tissue and promotes wound contraction, attenuating adverse remodelling.

### Expression analysis of some of the identified genes

**Figure 6:**
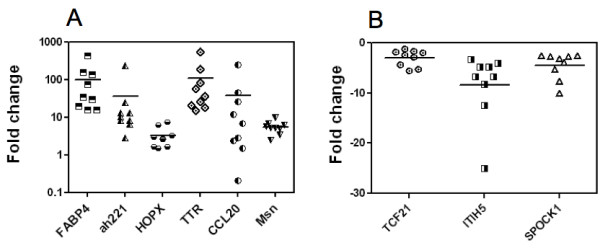
**Expression analyses of selected genes**. This figure shows the Quantitative Polymerase Chain Reaction (qPCR) of set of selected genes. FABP4, ah221, HOPX, TTR, CCL20, Msln, TCF21, ITIH5 and SPOCK1 gene expression was analyzed in 9 samples of wound and in the 9 corresponding controls. For each CAM the fold change in the wound was calculated from the ΔΔCt and plotted. We normalized these genes with the house keeping gene gHNRPH1 (Heterogeneous nuclear ribonucleoprotein H1) in all the tissues. A, upregulated genes; B, down-regulated genes.

The expression of 9 of the differentially regulated genes in the Affymetrix analysis was then investigated by quantitative real-time polymerase chain reaction (qRT-PCR) in wound tissue in comparison to its own control CAM (figure 6). We chose to analyze these genes because of their expression levels and because not much is known about their involvement in angiogenesis or tissue repair. Transcript levels, as determined by qPCR, were in accordance with the results obtained by the Affymetrix analysis. The respective expression levels were: FABP4 (100.5 fold), ah221 (35.4 fold), HOPX (3.3 fold), TTR (109 fold), CCL20 (37.5 fold), MSLN (5.5 fold), TCF21 (- 3 fold), ITH5 (-8.4 fold), and SPOCK1 (-4.5 fold). Control and wound samples from 9 eggs were analyzed.

**Figure 7:**
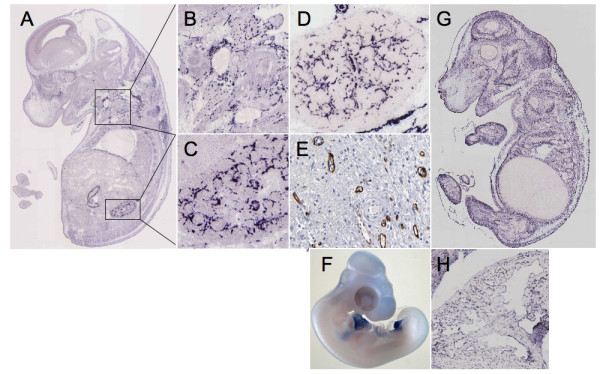
**Analysis of selected genes by in situ hybridization**. The expression patterns of FABP4, as analyzed by *in situ* hybridization of mouse E14.5 embryo, are displayed in panels A, B and C and the same for EMP-1 in panels F and G. Panels C and D, for direct comparison, show the expression patterns of FABP4 and CDH5 respectively in kidney. Panel E shows the expression profile of FABP4 in vessels of the urinary bladder as analyzed by immunohistochemistry. Panel G portrays the expression of EMP-1 in the endocard of an E14.5 embryo (*in situ* hybridization). Panel H depicts the expression of TCF21 in the E23 chick embryo (*in situ* hybridization).

We then analyzed expression of the different genes using GenePaint http://www.genepaint.org, ProteinAtlas http://www.proteinatlas.org and Geisha http://www.geisha.arizona.edu. In GenePaint, FABP4 was expressed in the vasculature in the stage 14.5 mouse embryo. Significant staining was seen in blood vessels (figure 7A to 7C), especially in the kidney (figure 7C). For comparison, the pattern of staining as seen in the kidney is identical to that of VE-Cadherin (CDH5), which is a specific marker of blood vessels (figure 7D). Furthermore, in the ProteinAtlas, staining of FABP4 was also seen in the vasculature of several organs such as the urinary bladder (figure 7E) but it is of note that not all blood vessels are stained for FABP4. There is currently only one publication describing the expression of FABP4 in the vasculature [28]. Our results are in agreement with these findings and indicate a strong expression of FABP4 after wounding. Chemokine ah221 is also significantly expressed after wounding. The human ortholog of ah221 is not yet identified but a similarity of 43% to human CCL3 is observed. CCL3 has leukocyte chemotactic activity and is involved in the recruitment of leucocytes and fibroblasts into neoangiogenic sites such as tumors [29]. CCL3 has also been shown to be produced by endothelial cells and to have an autocrine function [30]. It is likely that ah221 or its human ortholog has similar functions and plays a significant role in wound repair. HOPX is up-regulated 8.41 fold in our transcriptomic analysis. This gene is expressed during cardiac development, in various tumors, such as choriocarcinoma, and it is likely it may have a function in tissue repair as well [31,32].

Transthyretin (TTR) has been found highly up-regulated in our analysis. Transthyretin (TTR) is a plasma protein mostly known for being the transporter of thyroxine and retinol [33,34]. When mutated, TTR is also well-described as the cause of familial amyloid polyneuropathy, a neurodegenerative lethal disorder characterized by systemic deposition of TTR amyloid fibrils[34]. A potential role in tissue repair has not yet been described for this gene but it may have an indirect effect on tissue morphogenesis through retinol. This is supported by our IPA (Additional file 5, Network 6) where 15 genes up-regulated in the wound are known to be directly induced by retinoic acid.

CCL20 (MIP-3 α) is a CC chemokine family member that is highly expressed in our wound transcriptomic analysis. CCL20 activates CCR6 and leads to calcium mobilization and elevated active RhoA, phosphorylated myosin light chain, and F-actin accumulation and stimulation of epithelial cell migration [35]. Furthermore, in rat models of oral wound healing, CCL20 is up-regulated during the peak phase of wound healing [36]. These findings, together with ours, support an important role of this chemokine in wound healing.

Mesothelin (MSLN) is also significantly up-regulated after wounding. This is surprising since MSLN up-regulation is mainly found in mesothelioma, pancreatic, breast and ovarian carcinoma, and tumors of the GI tract http://www.proteinatlas.org. In normal tissue, significant expression is only observed in the fallopian tube http://www.proteinatlas.org. It has been shown that MSLN can interfere with cell cycle regulators by activating ERK kinase and decreasing BIM [37]. Furthermore, an increase in Stat3 activation and cyclin E in MSLN transfected pancreatic tumor cells is observed [38]. As in tumors, MSLN may promote GT formation by promoting proliferation of stromal fibroblasts and vascular cells.

TCF21, which is decreased by 3 fold in our qPCR analysis, has been implicated in kidney and lung organogenesis [39]. TCF21 -/- die in the early perinatal period because of multiple renal defects. TCF21 has also been described as a tumor suppressor gene that undergoes epigenetic modifications [40]. TCF21 has been implicated in myofibroblast differentiation and control of proliferation in mesenchymal progenitor cells [41]. During development, TCF21 is expressed at various sites in the chick embryo such as the pericardium or the allantois (figure 7H). During wound healing, TCF21 may be implicated in the regulation of fibroblast proliferation and differentiation in the GT. Inter-α globulin inhibitor 5 (ITIH5) decreased by 8.4 fold in our qPCR analysis, encodes one of the heavy chains of ITI, It is a protease inhibitor associated with the extracellular matrix and contributes to matrix stability by covalent linkage to hyaluronan. Loss of expression has been observed in various human solid tumors [42]. Furthermore, its loss by promoter hyper-methylation is associated with poor prognosis in mammary carcinoma [43]. During wound healing, this molecule is possibly involved in matrix remodeling. SPOCK1/SPARC/Osteonectin/testican-1 is decreased by 4.5 fold in our qPCR analysis. This molecule is a proteoglycan able to inhibit proteases such as MMP2 or Catepsin L [44]. SPOCK1 is also expressed in cancer associated fibroblasts where it reflects EMT [45]. However, SPOCK1 by itself inhibits angiogenesis, enhances tumor stroma formation and prevents fibroblast activation [46]. This may further explain its anti-tumor effect. Down-regulation of SPOCK1, as observed in our study, may contribute to an increase in vessel ingrowth and fibroblast activation during GT formation.

### In silico-endothelial data filtering

Herbert and coll. have developed a method to assign endothelial cell signatures to genes identified after gene profiling studies [47]. Endothelial cell data filtering evidenced several genes with significant endothelial cell signature (Table 4). For FABP4, despite the fact that endothelial transcript counts were not high, differences were significant because no counts were observed in the non-endothelial cell pool. As indicated previously, vascular endothelial cell expression of this molecule is very pronounced. For example, FABP4 is found in the vasculature at E14.5 (figure 7B).

**Table 4:** Endothelial cell data filtering

Gene	qvalue	Endo Count	Non-Endo	Endo factor	Up/Down	Angioscore	Fold change
EMP1	0.00	136	30	19.90593	Up	1	3.33
CD74	0.00	6	819	0.032169	down	10	6.28
MYCT1	0.00	16	0	∞	Up	0	0.4
CCL20	0.00	8	0	∞	Up	35	2.13
LCP1	0.00	1	60	0.073184	down	1	4.37
HLA-DPB1	0.00	0	42	0	down	2	4.56
PGD	0.00	13	10	5.708318	Up	10	2.52
GSTO1	0.00	14	13	4.728784	Up	2	4.3
LTA4H	0.00	9	5	7.903825	Up	0	2.55
FABP4	0.01	4	0	∞	Up	6	79.23
RGS1	0.01	4	0	∞	Up	6	9.28
CAP1	0.01	34	68	2.195507	Up	0	
MGST1	0.01	0	28	0	down	4	2.56
CD44	0.01	22	37	2.610873	Up	60	5.62

The table depicts a list of genes with positive Endo factor. Genes are listed according to q-values. Fold changes derived from the transcriptomic analysis and the Angioscore are also indicated.

The molecule with the highest endothelial transcript counts was epithelial membrane protein-1 (EMP1). EMP1 was induced in our transcriptomic analysis by about 3.3 fold in the GT after wounding. In GenePaint, expression of EMP1 was seen in vascular cells and possibly in the mesenchyme (figures 7F and 7G). Expression is much more diffuse than that of FABP4. EMP-1 is however, highly expressed in the endothelial cell lining of the endocard (figure 7G). There is only one single publication that reports EMP1 to be present at tight junctions in vascular endothelial cells [48]. This observation fits well with a role of EMP1 in tissue repair, since endothelial cell junctions are remodeled during endothelial cell migration in the GT.

Another gene highly expressed in endothelial cells was MYCT1. MYCT1 is a direct target of c-myc and phenocopies many of the effects of c-myc [49,50]. It has been described to be over-expressed in gastric carcinoma [51]. No publications with regard to endothelial cell expression of this gene have been reported, which is reflected by a surprisingly low Angioscore.

CCL20 exhibited a significant Endofactor and a high Angioscore. It has been reported that endothelial cells in culture express CCL20 upon thrombin stimulation [52]. CCL20 seems to be implicated in endothelial cell-lymphocyte interaction through CCR6 [53]. Lymphatic endothelial cells have been reported to express CCL20 upon induction by lipoteichoic acid (LTA) [54]. However, there have been reports where expression of CCL20 is outside the vasculature, such as in tumor cells [55]. The reason for these differences is not known but genome instability of tumor cells, leading to aberrant CCL20 expression, could be the reason.

### Integration into a general mechanism of wound repair and granulation tissue formation

From our results, some hypothesis can be formulated of how identified genes may fit into a general scheme of wound repair and GT formation. Tissue repair- is driven by positively and negatively acting factors. Early ingrowth of vessels and fibroblasts are driven through growth factors and cytokines such as IL8, IL1 and PDGFRB. Some additional chemokines including CCL20 or ah221 may also contribute to endothelial cell activation, vessel remodeling during wound healing and fibroblast recruitment. FABP4 may participate in wound repair by promoting endothelial cell proliferation. EMP1 may be involved in the modulation of intercellular adhesion in vessels after endothelial cell activation and participate in the mobility and sprouting of vessels in the GT.

The expression of several matrix or matrix-associated proteins (CL8A1, FBN1, Laminin-α1, FRAS-1, ITIH5 etc.) is likely to be modulated during wound repair and is decreased when significant GT formation had occurred. Down-regulation of SPOCK1, as observed in our study, may contribute to an increase in vessel in growth and fibroblast activation during GT formation. During wound healing, TCF21 may be implicated in the regulation of fibroblast proliferation and differentiation in the GT, and ITIH5 is possibly involved in matrix remodeling. Interferon-γ may interact with stroma fibroblasts and modify the cellular composition of the healing tissue, thus, promoting wound contraction, attenuating adverse effects on remodeling.

It is possible that vitamin A has a role in wound healing [56] as it is interesting that three genes out of the top four up-regulated in the wounded CAM (from 36 fold to 79 fold up-regulation) from this study are potentially retinol related (FABP4, RBP7 and TTR). It has been shown that TTR (Transthyretin) forms a complex with Retinol Binding Protein (potentially RBP7 here) for transport of retinol around the circulation [57]. In addition, retinol binding proteins have already been shown to be differentially expressed in GT[58]. Retinol is a precursor to retinoic acid, which acts as a steroid hormone, targeting nuclear receptors of genes involved in tissue morphogenesis [59]. It is possible this steroid hormone could be delivered to cells bound to a RBP7-TTR complex and be transported through the cell membrane by FABP4. FABP4 could also deliver retinoic acid to signalling molecules such as Retinoic acid receptors (RARs), Peroxisome Proliferator-Activated Receptors (PPARs) and nuclear response elements. Fatty acid binding proteins have been previously shown to do this [60]. This hypothesis is supported by previous work that found the topical addition of retinoic acid, derived from retinol, to genetically diabetic mice improves wound healing [61] and that corneal endothelial healing rates increase in the presence of retinoic acid[62]. The participation of other molecules such as Mesothelin is more difficult to envision because of lack of sufficient functional data.

There have been several studies that report transcriptomic profiling in wound tissue in different experimental settings. These include, for example, the transcriptome-wide analysis in excisional murine cutaneous wound inflammation [63]or in chronic ischemic wounds in the pig model [64]. These studies are different to ours because they are performed in an immunocompetent setting and, thus, do not address exclusively the role of the stromal fibroblasts and blood vessels. There has been one study that performed a transcriptome-wide analysis of blood vessels laser captured from human skin and chronic wound-edge tissue [65]. However, in this case contamination by circulating mononuclear cells can also not not be excluded. There have also been transcriptomic profiling studies using models of tissue regeneration such as regeneration of *Xenopus laevis *hindlimbs[66]or fin regeneration in the medaka fish [67]. These studies are different from ours because myofibroblast invasion does not occur in these models. Our study is complementary to these existing transcriptome profling studies and provides additional informations on the gene networks implicated in wound repair and GT formation.

## Conclusion

The CAM wound model has been established to analyze GT formation and the role of invading fibroblasts and blood vessels in this process [10]. It has been found that tissue tension generated by activated fibroblasts or myofibroblasts during wound contraction, mediated and directed translocation of the vasculature. This vasculature can be expanded, secondarily by elongation and vessel enlargement, and finally through splitting and sprouting. We report herein a complete transcriptome analysis of the "wound model" in the chicken CAM, which allowed the identification of gene signatures involved in GT formation and neoangiogenesis. Cytokines and chemokines clearly play a central role as evidenced in our analysis. The limitation of our work is that, contrary to the adult organism, our model is devoid of immunocompetent cells[68]. However, it has been described that MMP-9 positive neutrophile-like inflammatory cells and MMP-13 positive monocyte-like cells are accumulated in growth factor-stimulated CAM[9]. Thus, these cells may also participate, besides blood vessels and stromal fibroblasts, in GT formation after wounding in the CAM.

Another possible limitation is the relevance of our findings for the mammalian setting. Indeed, it is known that some of the regulators identified in the mammalian system that are involved in vascular development are not present in the chick such as the VE-statins [69]. However, this is a general problem for every model organism including murin models. As an example, CXCL4L1 is only expressed in man, mouse and chimpanzee. CXCL4L1 is a potent angio-inhibitory chemokine that has potent inhibitory activity across species. Furthermore, our laboratory has performed molecular profiling studies using human xenograft tissue in the chick CAM and identified gene regulatory mechanisms relevant for the mammalian setting [12,70]. Thus, we believe, that our results are of importance to the general understanding of GT formation and tissue repair.

## Methods

### Tissue wounding

Brown Leghorn eggs were cultured at 38°C for 3 days. The shells were then cracked and the contents transferred to 10 cm cell-culture Petri dishes. Embryo culture was continued for a another 7 days, when two injuries to the CAM were inflicted by parallel scalpel superficial cuts of 1 cm area, with a subsequent scarping off the epithelium of the injured chorioallantoic membrane. The wound area was then covered with 1.5 cm square nylon grid and after 6 days, the CAM tissue (control and wound) were excised and processed for subsequent analysis.

### RNA isolation

Total RNA from cells or snap-frozen tissues were extracted by using RNeasy mini kit (Qiagen, Courtaboeuf, France). RNA quality and quantity were assessed by agarose gel electrophoresis and optical density measurement. First strand cDNA was prepared from 1 μg of total RNA with Quantitect Reverse Transcription kit (Qiagen). For all samples, a negative control was realized with mRNA without reverse transcriptase in the reaction mixture.

RNA was isolated from control and "wound" CAM 6 days after injury. Three eggs were used for independent transcriptomic profiling. It is important to note that from each egg, unwounded control CAM and wound were analyzed. RNA was isolated according to standard procedures and hybridized to Affymetrix chicken GeneChips using the Affymetrix standard protocol (Affymetrix UK Ltd, High Wycombe, UK).

### Transcriptome analysis

RNAs hybridized to Affymetrix chicken GeneChips using the Affymetrix standard protocol (Affymetrix UK Ltd, High Wycombe, UK). The chicken GeneChip covers 32773 transcripts, corresponding to > 28000 chicken genes, and has a probe set oligonucleotide length of 25 and a detection sensitivity of 1:100000 http://www.affymetrix.com. Data were analyzed with the GCOS 1.2 software (Affymetrix), using the default analysis settings; global scaling as first normalization method, with a trimmed mean target intensity value (TGT) of each array arbitrarily set to 100.

Gene expression profiles were identified using two-class Significance Analysis of Microarrays (SAM) method [71] (http://www-stat.stanford.edu/~tibs/SAM/, which utilizes a Wilcoxon-test statistic and sample-label permutation to evaluate statistical significance between sample groups. SAM provides mean fold change values (FC) (mean fold-change > 2) and a false discovery rate (FDR) confidence percentage based on data permutation (n = 200). The False Discovery Rate (FDR), an estimate of the fraction of selective genes, was kept below 5% in all statistical analyses.

Data analysis was done using the Gene ontology database included in the statistical environment R library GOstat http://www.geneontology.org and Ingenuity Pathway Analysis (IPA) (Ingenuity Systems, Redwood City, CA 94063) software. The functional clustering was performed using the method implemented on the DAVID website http://david.abcc.ncifcrf.gov/.

Annotation of genes was performed using NetAffx http://www.affymetrix.com. The microarray data files have be submitted to the US National Center for Biotechnology Information, Gene Expression Omnibus (GEO), and released in May 6^th^, 2010 (GEO accession number: GSE21679).

### qPCR analysis

Real-time PCR was carried out in an Mx3000P thermocycler (Stratagene, La Jolla, CA) by using SYBR Green dye (ABgene, Courtaboeuf, France). Chicken-specific primers were designed and respectively evaluated for amplification efficiency using total RNA isolated from a total chicken embryo on embryonic day 5. Only primers pairs with amplification efficacy between 90 and 100% were used. The PCR specificity was verified by dissociation curve analysis and agarose gel electrophoresis of the amplification product.

The primer sequences are: gah221 forward CTGGCCCTCTGCTCCTCA and reverse GGACGGGACGTTGAACATAG, gCCL20 forward CGGAAGGTCATTAAGGGC and reverse AAACCATATCACATTGACATCCTC, gFABP4 forward AGACTGCTACCTGGCCTGAC and reverse GCCATCTTCCTGGTAGCAAA, gHOPX forward GCAGTCACGCTGGCTATAAA and reverse CCATTTCTCCTGGATGGTG, gITIH5 forward TCTTGTTGCCCTTGGAAATC and reverse TTCTTTCCTCCCACCTCCTT, gMsln forward AAAATGAACAGGCTGCTGCT and reverse TCAGGCTGTTGGGGTCTATC, gSPOCK1 forward AAAGCAGGGGACCGTTAGTT and reverse TTCCAAATCATCCAGCAACA, gTCF21 forward CCATCCAGTCAACCTGACCT and reverse AGCGGTTTGTGTTCACCACT, gTTR forward TTGATTCCAAATGCCCTCTC and reverse TAGCAAAGTCCTGCCAGGTT and the house keeping gene gHNRPH1 forward GCTGTGTCTGCCACGAGTTA and reverse GCTTTCGGCTGAGAGACAAT.

### Predicting human ortholog of chicken genes

To concentrate on genes of importance to human pathology and physiology, human orthologs of the chicken genes present on the Affymetrix chicken GeneChips were predicted using a Reciprocal Best Hit (RBH) approach[72]. In this work, both human and chicken Refseq nucleotide and protein sequences were downloaded from the NCBI on 30^th^ January 2008 ftp://ftp.ncbi.nih.gov/refseq. Likewise, cDNA accession numbers of sequences used to design the microarray probes were extracted from Affymetrix chicken chip file "Chicken.na22.annot.csv" http://www.affymetrix.com. Each cDNA sequence, depending on the source of the probe design, was downloaded from ENSEMBL or the NCBI, and, as most of the cDNA sources were Expressed Sequence Tags, full length chicken mRNAs were sought by BLAST searching each of them against the Refseq chicken nucleotide database. The resulting matches were ranked as good, reasonable or bad, depending on the alignment quality (Good: sequence alignment > 100 base pairs with a percent identity > = 96%. Reasonable: a sequence alignment > 100 bases and a percent identity > 90% and < = 95%. Bad: all other hits). The full length chicken mRNA sequences ranked good and reasonable were then used in a RBH analysis.

### Defining chicken endothelial cell genes using human orthologs and human cDNA library analyses

To identify which chicken genes could have an endothelial cell expression signature, the human orthologs were compared with the results from a novel *in-silico* bioinformatics screen, where an accurate EST-to-gene assignment and a new likelihood ratio statistic were used to find genes preferentially expressed in endothelial cells using cDNA library analyses (see Herbert et al. 2008 for a full description). The intersection of the comparison were endothelial genes and only those genes [47] with a q-value < = 0.01 were considered. An "Endofactor" describes how significant a gene was endothelial as found with the q-value.

### Literature scanning of human-chick orthologs: "Angioscore"

For all the genes found differentially expressed on the chicken chip, a literature search of the human orthologs of chicken genes were carried out to find those having literature relating to relevant pathologies and physiologies. To accomplish this, Perl scripts were written that searched article abstracts for the following keywords typical of angiogenic research. They were "angiogenic", "angiogenesis", "neovascularis(z)ation", "vasculogenesis", "vascular", "VEGF", "hypoxia" and "endoth" (for endothelial or endothelium).

### Expression analysis

In situ hybridization and immunostaining data were retrieved from the Genepaint data base (Max-Planck-Institute of Biophysical Chemistry, Dept. Genes and Behavior, 37077 Goettingen

Germany; http://www.genepaint.org; figure 7A to 7D, F and 7G), Proteinatlas (AlbaNova University Center at the Royal Institute of Technology, Stockholm, Sweden, the Rudbeck Laboratory, Uppsala University, Uppsala, Sweden and Lab Surgpath, Mumbai, India; http://www.proteinatlas.org; figure 7E) and the GEISHA database (University of Arizona, Tucson, AZ 8572; http://geisha.arizona.edu[73]; figure 7F).

### Histology and Immunohistology

Wound areas were fixed for at least 24 hours in a Zn-fixative [74]. Tissues were embedded in paraplast, and section of 10 μm with a MICROM HM325 were performed and placed onto Super Frost slides. Dewaxed slides were either stained with Weigert's hematoxylin and eosin or incubated 40 min at 95°C in a citrate buffer for the antigen recovery before immunohistochemistry. These slides were then fixed with 4% paraformaldehyde, permeabilized with Triton-X100 (0.1%), saturated with 5% BSA in PBS (pH 7.4) and incubated with the primary antibody over-night at 4°C (anti-human Smooth Muscle actin, DAKO, IR611) This antibody also recognizes very well the chicken protein. Secondary fluorescent anti-mouse antibody was from Molecular Probes (used at1:1,000, Invitrogen). Chick blood vessels were visualized by using fluorescein-coupled *Sambucus nigra* lectin (SNA-1 lectin, 1:100, Vector Laboratories). Cell nuclei were visualized by DAPI (Invitrogen). Microphotographs were taken with a Nikon eclipse E600 microscope equipped with a digital camera Nikon DS-Ri1.

## List of abbreviations

ATOH8: Atonal hololog 8; CCL: chemokine (CC motif) ligand; CXCL: chemokine (C-X-C motif) ligand; CAM: chorioallantoic membrane; C1q: Complement factor 1q; CRISP3: cysteine-riche secretory protein; EMP1: epithelial membrane protein-1; FABP4: fatty acid binding protein-4; GT: granulation tissue; HOPX: Homeobox PX; ITIH5: inter-a (globulin) inhibitor H5; LAMA1: laminin a1; LTA: lymphotoxin alpha; MSLN: mesothelin; MYCT1: myc target-1; NCF2: neutrophile cytosolic factor; PLXNA2: plexin A2; RBH: reciprocal Best Hit; RTBP7: retinol binding protein 7; SPP1: osteopontin; SPOCK1: sparc/osteonectin, cwcv and kazal-like domains proteoglycan (testican); C1QTNF1: TNF related factor-1; TCF21: transcription factor 21; TTR: transthyretin; TGT: trimmed mean target intensity value.

## Authors' contributions

WK did CAM experiments, FS did expression analysis, PA and FF did bioinformatics analysis, JH and RB did the endothelial and Angioscore screens, AB supervised the work and wrote the manuscript. All authors read and approved the final manuscript.

## Authors' information

WK and FS have been post-doctoral fellows in the AB laboratory, PA is PhD student in the FF laboratory, JH is bioinformatics officer in the RB laboratory, RB is Professor in the division of Immunity and Infection at the Institute for Biomedical Research at the University of Birmingham Medical School (UK), FF is senior lecturer at the School of Biosciences at the University of Birmingham (UK), AB is Professor in cell and molecular biology at the university Bordeaux and director of the molecular angiogenesis laboratory of the National Institute for Health and Medical Research (INSERM, France).

## Supplementary Material

Additional file 1 **(additional Table S1): Clustering of genes with similar function according to Gene Ontology terms**. Click here for file

Additional file 2 **(additional Table S2): List of all up-regulated genes (cut off > 2)**. Click here for file

Additional file 3 **(additional Table S3): List of all down-regulated genes (cut off > 0.1)**. Click here for file

Additional file 4 **(additional Table S4): Ingenuity pathway analysis**. Regulated genes were analyzed by Ingenuity pathway analysis (IPA) in order to determine functional categories. Functional categories are listed in the tables that depict categories for up-regulated (A) or down-regulated genes (B). As it can been seen in the tables, categories related to tissue morphological processes have the highest Ingenuity scores. Click here for file

Additional file 5 **(additional Figure S1A-J): Representation of networks from the IPA for up-(A-H) or down-regulated genes (I-J)**. Click here for file
