# Supplementary material for: Gene signatures in wound tissue as evidenced by molecular profiling in the chick embryo model
Source: BMC Genomics. 2010 Sep 14;11:495. doi: 10.1186/1471-2164-11-495 (PMC2996991; doi:10.1186/1471-2164-11-495)
Supplement: Additional file 2 — (additional Table S2): List of all up-regulated genes (cut off > 2). [file 1471-2164-11-495-S2.PDF]

Affymetrix ID	UniGene ID	Gene Name	Gene Symbol	Fold Change
Gga.4939.1.S1_s_at	Gga.4939	fatty acid binding protein 4, adipocyte	FABP4	79.23
Gga.9386.1.S1_at	Gga.9386	retinol binding protein 7, cellular	RBP7	73.82
Gga.17686.1.S1_at	Gga.41504	keratin 75	KRT75	59.38
Gga.2620.1.S1_at	Gga.2620	transthyretin	TTR	36.7
Gga.3551.1.S1_at	Gga.3551	secreted phosphoprotein 1 (osteopontin, bone sialoprotein I, early T-lymphocyte activation 1)	SPP1	30.01
Gga.17647.1.S1_at	Gga.34552	neutrophil cytosolic factor 2 (65kDa, chronic granulomatous disease, autosomal 2)	NCF2	25.09
Gga.7228.1.S1_at	Gga.7228	carboxymethylenebutenolidase homolog (Pseudomonas)	CMBL	14.33
Gga.11640.1.S1_at	Gga.11640	succinate receptor 1	SUCNR1	11.04
GgaAffx.10393.1.S1_at	Gga.46851	cytochrome b-245, beta polypeptide (chronic granulomatous disease)	CYBB	10.25
Gga.9133.1.S1_at	Gga.9133	Chemokine ah221	LOC417536	9.95
Gga.6239.1.S1_at	Gga.6239	regulator of G-protein signalling 1	RGS1	9.28
Gga.11456.1.S1_at	Gga.11456	cystatin A (stefin A)	CSTA	9.25
Gga.3383.1.S1_at	Gga.3383	lipopolysaccharide-induced TNF factor	LITAF	9.16
Gga.729.1.S1_at	Gga.729	mature avidin	LOC396260	8.41
Gga.1158.3.S1_a_at	Gga.1158	HOP homeobox	HOPX	8.41
GgaAffx.6592.1.S1_at	---	---	---	8.36
GgaAffx.25031.1.S1_at	Gga.19498	cysteine-rich secretory protein 3	CRISP3	8.29
Gga.17679.1.S1_s_at	Gga.39008	similar to immunoglobulin-like receptor CHIR-AB3 -B4 -B5 -B	LOC425449	8.2
GgaAffx.11785.1.S1_s_at	Gga.9879	lipase A, lysosomal acid, cholesterol esterase (Wolman disease)	LIPA	8.17
Gga.5743.1.S1_at	Gga.5743	lymphocyte antigen 96	LY96	8.09
GgaAffx.8148.1.S1_at	Gga.41242	serpin peptidase inhibitor, clade B (ovalbumin), member 5	SERPINB5	7.19
GgaAffx.20689.1.S1_at	Gga.1313	spleen focus forming virus (SFFV) proviral integration oncogene spi1	SPI1	7.11
Gga.11539.1.S1_at	Gga.46810	Similar to interferon, gamma-inducible protein 30	LOC420129	6.86
Gga.6991.2.S1_a_at	Gga.6991	Transcribed locus	---	6.8
GgaAffx.22156.1.S1_at	---	---	---	6.61
Gga.5396.1.S1_at	Gga.5396	complement component 1, q subcomponent, B chain	C1QB	6.61
Gga.703.1.S1_at	Gga.703	leukocyte ribonuclease A-1	LOC396194	6.57

GgaAffx.3346.1.S1_at	Gga.33177	mesothelin	MSLN	6.49
Gga.6332.1.S1_at	Gga.6332	similar to complement regulatory soluble protein	LOC771877	6.34
Gga.4414.1.S1_s_at	Gga.4414	CD74 molecule, major histocompatibility complex, class II invariant chain	CD74	6.28
Gga.7159.1.S1_at	Gga.7159	lysosomal associated multispanning membrane protein 5	LAPTM5	6.16
Gga.7838.1.S1_at	Gga.30124	colony stimulating factor 2 receptor, alpha, low-affinity (granulocyte-macrophage)	CSF2RA	6.06
Gga.6390.3.S1_a_at	Gga.6390	cathepsin C	CTSC	5.65
Gga.3572.1.S1_at	Gga.3572	CD44 molecule (Indian blood group)	CD44	5.62
GgaAffx.8414.2.S1_s_at	Gga.22164	complement component 3a receptor 1	C3AR1	5.6
Gga.12420.1.S1_at	---	---	---	5.56
GgaAffx.3506.1.S1_at	Gga.28353	ecotropic viral integration site 2B	EVI2B	5.39
Gga.8164.1.S1_a_at	Gga.8164	similar to Ras-related protein Rab-10	LOC421099	5.32
Gga.9880.1.S1_at	Gga.37358	Purinergic receptor P2Y, G-protein coupled, 13	P2RY13	5.32
Gga.10971.1.A1_at	---	Xg blood group	XG	5.28
Gga.1951.1.S1_at	Gga.1951	gap junction protein, beta 6, 30kDa	GJB6	5.25
Gga.4507.1.S1_at	Gga.4507	integrin, beta 2 (complement component 3 receptor 3 and 4 subunit)	ITGB2	5.24
Gga.5164.1.S2_at	Gga.5164	BCL2-related protein A1	BCL2A1	5.15
Gga.14969.1.S1_at	Gga.14969	Abhydrolase domain containing 7	ABHD7	4.89
GgaAffx.26062.1.S1_s_at	---	similar to immunoglobulin-like receptor CHIR-B3	LOC426119 /// LOC770720	4.78
GgaAffx.11801.1.S1_at	Gga.42499	synaptogyrin 3	SYNGR3	4.76
Gga.969.1.S1_at	Gga.969	monooxygenase, DBH-like 1	MOXD1	4.67
Gga.4416.1.S1_at	Gga.4416	serpin peptidase inhibitor, clade I (neuroserpin), member 1	SERPINI1	4.65
GgaAffx.8892.1.S1_at	Gga.39541	similar to immunoglobulin-like receptor CHIR-A2	LOC426330	4.61
GgaAffx.3968.1.S1_at	Gga.29543	hypothetical LOC424855	LOC424855	4.58
Gga.13336.5.S1_x_at	Gga.42424	MHC class II beta chain /// MHC class II antigen B-F minor heavy chain	BLB1 /// BLB2	4.56
Gga.11227.1.S1_at	Gga.25798	bone marrow stromal cell antigen 1	BST1	4.5
Gga.4719.1.S1_s_at	Gga.34313	apolipoprotein A-I	APOA1	4.4
GgaAffx.26327.1.S1_x_at	Gga.38935	similar to immunoglobulin-like	LOC425309	4.38

		receptor CHIR-AB3 -B -B5 -B4		
GgaAffx.10876.2.S1_s_at	Gga.22088	lymphocyte cytosolic protein 1 (L-plastin)	LCP1	4.37
Gga.6848.2.S1_at	Gga.6848	GLI pathogenesis-related 1 (glioma)	RCJMB04_20n13	4.37
Gga.6650.3.S1_a_at	Gga.41869	similar to programmed death ligand 1	LOC427225	4.36
Gga.5507.1.S1_at	Gga.10968	glutathione S-transferase omega 1	GSTO1	4.3
Gga.21.1.S1_a_at	Gga.21	interleukin 2 receptor, gamma (severe combined immunodeficiency)	IL2RG	4.27
GgaAffx.12323.1.S1_s_at	Gga.34664	ecotropic viral integration site 2A	EVI2A	4.26
GgaAffx.23262.1.S1_at	Gga.10465	G protein-coupled receptor 65	GPR65	4.25
GgaAffx.10696.1.S1_at	Gga.24899	phosphatidylinositol-specific phospholipase C, X domain containing 1	PLCXD1	4.24
Gga.7298.1.S1_at	Gga.7298	complement component 1, q subcomponent, A chain	C1QA	4.2
Gga.6529.1.S1_at	Gga.6529	Fc fragment of IgE, high affinity I, receptor for; gamma polypeptide	FCER1G	4.12
Gga.5568.1.S1_at	Gga.5568	Finished cDNA, clone ChEST576b15	---	4.01
Gga.8752.1.S1_s_at	Gga.8752	sorting nexin 10	SNX10	4.01
Gga.9873.1.S1_at	Gga.9873	complement component 1, q subcomponent, C chain	C1QC	4.01
Gga.10034.2.S1_s_at	Gga.10034	serpin peptidase inhibitor, clade B (ovalbumin), member 2	SERPINB2	3.97
GgaAffx.12715.1.S1_s_at	Gga.22398	glia maturation factor, beta	RCJMB04_21j8	3.93
Gga.2039.1.S1_at	Gga.2039	heme oxygenase (decycling) 1	HMOX1	3.92
GgaAffx.6896.1.S1_at	---	similar to RASGEF1B protein	LOC776184	3.86
Gga.1193.1.S2_at	Gga.1193	protein tyrosine phosphatase, receptor type, C	PTPRC	3.83
Gga.9935.1.S1_at	Gga.45540	deleted in malignant brain tumors 1	DMBT1	3.81
Gga.16689.1.S1_s_at	Gga.30272	parvin, beta	PARVB	3.81
Gga.13484.1.S1_at	Gga.42186	interleukin 15	IL15	3.78
Gga.871.1.S1_at	Gga.871	solute carrier family 2 (facilitated glucose transporter), member 3	SLC2A3	3.77
Gga.7788.1.S1_at	Gga.7788	similar to phospholipase A2 inhibitor gamma subunit B	LOC426893	3.76
Gga.826.1.S1_s_at	Gga.826	interleukin 8	IL8	3.72
GgaAffx.12983.1.S1_at	Gga.43690	glucuronidase, beta	RCJMB04_28l23	3.72
Gga.6025.1.S1_s_at	Gga.6025	cathepsin S	CTSS	3.66
Gga.6093.1.S1_at	Gga.6093	chromosome 9 open reading frame 91	C9orf91	3.64
GgaAffx.12658.1.S1_at	Gga.21246	neutrophil cytosolic factor 4,	NCF4	3.63

GgaAffx.12658.1.S1_at	Gga.21246	neutrophil cytosolic factor 4, 40kDa	NCF4	3.63
Gga.11016.1.S1_at	Gga.11016	B locus M alpha chain 1	B-MA1	3.6
Gga.9362.2.S1_at	Gga.9362	hypothetical LOC423783	LOC423783	3.59
GgaAffx.10679.1.S1_at	Gga.24853	similar to GM-CSF receptor	LOC418667	3.59
Gga.15142.1.S1_s_at	Gga.3137	prostaglandin-D synthase	PGDS	3.57
GgaAffx.6177.1.S1_at	Gga.24203	glucosamine (N-acetyl)-6-sulfatase (Sanfilippo disease IIID)	GNS	3.57
GgaAffx.25280.1.S1_at	Gga.3602	coactosin-like 1 (Dictyostelium)	COTL1	3.55
Gga.9109.1.S1_at	Gga.9109	Transcribed locus	---	3.5
Gga.1294.1.S1_at	Gga.1294	myosin IF	MYO1F	3.49
Gga.236.1.S1_at	Gga.236	lymphocyte cytosolic protein 2 (SH2 domain containing leukocyte protein of 76kDa)	LCP2	3.49
Gga.7046.1.S1_s_at	Gga.7046	Deleted in malignant brain tumors 1	DMBT1	3.48
Gga.7176.1.S1_at	Gga.7176	mitochondrial trans-2-enoyl-CoA reductase	MECR	3.39
Gga.5393.1.S1_at	Gga.5393	similar to serine protease	LOC423941	3.35
Gga.9261.1.S1_a_at	Gga.9261	sclerostin domain containing 1	SOSTDC1	3.34
Gga.1939.1.S1_at	Gga.8392	Epithelial membrane protein 1	EMP1	3.33
Gga.6666.1.S1_at	Gga.6666	similar to serine protease inhibitor Kazal type 9	LOC770450	3.29
Gga.11395.1.S1_at	Gga.11395	G protein-coupled receptor 34	GPR34	3.27
Gga.12236.1.S1_at	Gga.12236	Transcribed locus	---	3.26
Gga.4070.1.S1_at	Gga.19453	Colony stimulating factor 1 receptor, formerly McDonough feline sarcoma viral (v-fms) oncogene homolog	CSF1R	3.25
Gga.2533.1.S1_s_at	Gga.2533	glutathione S-transferase A1	GSTA1	3.24
Gga.6064.1.S1_at	---	---	---	3.2
Gga.2408.1.S1_at	Gga.6577	branched chain aminotransferase 1, cytosolic	BCAT1	3.18
Gga.15768.1.S1_at	Gga.32370	tumor necrosis factor, alpha-induced protein 6	TNFAIP6	3.15
Gga.11614.1.S1_at	Gga.1180	serpin peptidase inhibitor, clade F (alpha-2 antiplasmin, pigment epithelium derived factor), member 1/2	SERPINF1 /// SERPINF2	3.13
Gga.11658.1.S1_at	Gga.11658	bactericidal/permeability-increasing protein	BPI	3.13
GgaAffx.11201.3.S1_s_at	Gga.37297	toll-like receptor 6 /// similar to toll-like receptor 1 /// Toll-like receptor 1 type 2 /// toll-like receptor 16	CHTLR1-TYPE2 /// TLR16 /// TLR6	3.1
Gga.12604.1.S1_a_at	Gga.12604	membrane-spanning 4-domains, subfamily A, member 15	MS4A15	3.07

Gga.3095.1.S1_a_at	Gga.3095	similar to hypothetical protein FLJ22662	LOC417967	3.06
GgaAffx.1342.1.S1_at	Gga.28157	zinc finger CCCH-type containing 12A	ZC3H12A	3.01
Gga.17090.1.S1_s_at	Gga.17090	scinderin	SCIN	3.01
Gga.20069.1.S1_at	Gga.38662	Finished cDNA, clone ChEST1021j1	---	2.98
Gga.7352.1.S1_at	---	---	---	2.94
GgaAffx.11658.1.S1_s_at	Gga.22314	neutrophil cytosolic factor 1	RCJMB04_3f13	2.9
GgaAffx.25386.2.S1_s_at	Gga.18555	dedicator of cytokinesis 2	DOCK2	2.89
Gga.1111.1.S1_a_at	Gga.1111	ubiquitin specific peptidase 18	USP18	2.89
Gga.10748.1.S1_at	---	---	---	2.88
Gga.17521.1.S1_s_at	Gga.17521	N-acylsphingosine amidohydrolase (acid ceramidase) 1	ASAH1	2.88
GgaAffx.26115.1.A1_at	Gga.16606	hypothetical gene supported by CR387553	LOC424026	2.87
GgaAffx.9441.2.S1_s_at	Gga.22533	Similar to Sphingomyelin phosphodiesterase, acid-like 3A	LOC776223	2.86
Gga.4772.1.S1_at	Gga.4772	Finished cDNA, clone ChEST662i24	---	2.86
Gga.7625.1.S1_at	Gga.36902	Finished cDNA, clone ChEST293h11	---	2.85
Gga.12980.1.S1_s_at	Gga.3447	phospholipase C, delta 1	PLCD1	2.82
GgaAffx.3452.1.S1_at	Gga.22736	phosphoinositide-3-kinase, catalytic, beta polypeptide	PIK3CB	2.81
Gga.10753.1.S1_at	Gga.10753	receptor accessory protein 3	REEP3	2.81
GgaAffx.20260.1.S1_at	Gga.34896	Finished cDNA, clone ChEST806n6	---	2.79
GgaAffx.21123.1.S1_s_at	Gga.3854	cathepsin B	CTSB	2.78
Gga.4083.1.S1_at	Gga.4083	homeodomain protein	NKX-6.1	2.71
Gga.198.1.S1_at	Gga.198	matrix metallopeptidase 9 (gelatinase B, 92kDa gelatinase, 92kDa type IV collagenase)	MMP9	2.67
Gga.11825.1.S1_s_at	Gga.11825	glycerol kinase	GK	2.67
GgaAffx.22812.1.S1_at	Gga.44425	hypothetical LOC426902	LOC426902	2.67
GgaAffx.22242.2.S1_s_at	---	---	---	2.66
Gga.6492.1.S1_at	Gga.6492	similar to chemokine CXCL13/BCA-1	LOC422510	2.66
GgaAffx.23937.1.S1_s_at	Gga.4462	myosin, heavy chain 9, non-muscle	MYH9	2.63
GgaAffx.11975.1.S1_s_at	Gga.4403	prosaposin	PSAP	2.63
Gga.2149.1.S1_s_at	Gga.2149	UDP-GlcNAc:betaGal beta-1,3-N-acetylglucosaminyltransferase 2	B3GNT2	2.61
GgaAffx.23416.1.S1_at	Gga.29461	solute carrier family 6, member 15	SLC6A15	2.6

Gga.12042.1.S1_at	Gga.12042	Interleukin 1 receptor accessory protein	IL1RAP	2.6
GgaAffx.20296.1.S1_at	Gga.17550	Similar to similar to 60 kDa heat shock protein, mitochondrial precursor (Hsp60) (60 kDa chaperonin) (CPN60) (Mitochondrial matrix protein P1) (HSP-65)	LOC777450	2.6
Gga.6031.1.S1_s_at	Gga.6031	hepatitis A virus cellular receptor 1	RCJMB04_10h2	2.59
Gga.7092.1.S1_at	Gga.7092	similar to FLJ00156 protein	LOC423781	2.59
Gga.13994.1.S1_at	Gga.13994	secretory carrier membrane protein 1	SCAMP1	2.59
GgaAffx.21502.1.S1_at	Gga.28486	UDP-GlcNAc:betaGal beta-1,3-N-acetylglucosaminyltransferase 5	B3GNT5	2.59
GgaAffx.4586.1.S1_at	Gga.8861	mitogen-activated protein kinase kinase kinase 8	MAP3K8	2.57
Gga.6261.1.S1_at	Gga.38109	microsomal glutathione S-transferase 1	MGST1	2.56
Gga.7082.1.S1_at	Gga.34748	Finished cDNA, clone ChEST79i3	---	2.56
Gga.11612.1.S1_s_at	Gga.11612	leukotriene A4 hydrolase	LTA4H	2.55
GgaAffx.12483.1.S1_at	Gga.21399	serum/glucocorticoid regulated kinase family, member 3	SGK3	2.55
GgaAffx.9516.1.S1_at	Gga.22196	coagulation factor II (thrombin) receptor-like 1	F2RL1	2.54
Gga.998.2.S1_a_at	Gga.998	CD3d molecule, delta (CD3-TCR complex)	CD3D	2.54
Gga.8635.1.S1_at	Gga.8594	solute carrier family 5 (sodium/glucose cotransporter), member 1	SLC5A1	2.54
Gga.3723.1.S1_at	Gga.3723	phospholipase A2, group IVA (cytosolic, calcium-dependent)	PLA2G4A	2.54
GgaAffx.8725.1.S1_s_at	Gga.22514	CNDP dipeptidase 2 (metallopeptidase M20 family)	CNDP2	2.53
Gga.19451.1.S1_at	Gga.19451	ADAM metallopeptidase domain 28	RCJMB04_17j13	2.53
Gga.7158.1.S1_at	Gga.7158	CD164 molecule, sialomucin	CD164	2.53
GgaAffx.12747.1.S1_s_at	Gga.1282	phosphogluconate dehydrogenase	PGD	2.52
GgaAffx.7309.2.S1_s_at	Gga.40185	nucleotide-binding oligomerization domain containing 1	NOD1	2.5
Gga.8259.1.S1_at	Gga.37229	Megakaryocyte-associated tyrosine kinase	MATK	2.48
GgaAffx.4379.1.S1_at	Gga.32116	toll-like receptor 4	TLR4	2.48
Gga.4398.1.S1_at	Gga.4398	lactate dehydrogenase A	LDHA	2.48
GgaAffx.7796.1.S1_at	Gga.23156	solute carrier family 39 (zinc transporter), member 8	SLC39A8	2.47

Gga.19327.1.S1_at	Gga.35778	Finished cDNA, clone ChEST912j10	---	2.46
GgaAffx.12646.1.S1_at	Gga.22529	spleen tyrosine kinase	RCJMB04_19o18	2.46
Gga.4503.1.S1_a_at	Gga.8606	phosphoglycerate kinase 1	PGK1	2.45
Gga.9486.1.S1_s_at	Gga.9486	La ribonucleoprotein domain family, member 5	LARP5	2.45
Gga.1712.1.S1_a_at	Gga.1712	ATPase, H+ transporting, lysosomal 70kDa, V1 subunit A	ATP6V1A	2.45
Gga.16552.2.S1_a_at	Gga.16552	hypothetical gene supported by CR387685	LOC421780	2.45
Gga.5761.1.S1_at	Gga.5761	EF-hand domain family, member D1	EFHD1	2.45
Gga.19.1.S1_at	Gga.19	interleukin 1, beta	IL1B	2.44
GgaAffx.21769.1.S1_s_at	Gga.1152	lipoprotein lipase	LPL	2.43
Gga.2689.2.S1_s_at	Gga.23765	acid phosphatase 1, soluble	ACP1	2.43
Gga.8312.4.S1_a_at	Gga.42199	similar to thioesterase B	LOC769339	2.41
GgaAffx.2281.1.S1_at	Gga.41699	unc-119 homolog (C. elegans)	UNC119	2.41
GgaAffx.181.1.S1_at	Gga.40595	plakophilin 1 (ectodermal dysplasia/skin fragility syndrome)	PKP1	2.4
Gga.13407.1.S1_at	Gga.22955	Nedd4 family interacting protein 2	NDFIP2	2.4
GgaAffx.2598.1.S1_at	Gga.7080	sterol O-acyltransferase (acyl-Coenzyme A: cholesterol acyltransferase) 1	SOAT1	2.4
GgaAffx.2750.1.S1_s_at	Gga.7113	RAB27A, member RAS oncogene family	RAB27A	2.39
Gga.701.1.S1_s_at	Gga.34358	leukocyte ribonuclease A-1 /// leukocyte ribonuclease A-2	RSFR	2.39
GgaAffx.13083.1.S1_s_at	Gga.11550	family with sequence similarity 49, member A	FAM49A	2.37
Gga.15971.1.S1_at	Gga.15971	Finished cDNA, clone ChEST136j22	---	2.37
Gga.11955.1.S1_at	Gga.36548	Immunoglobulin superfamily, member 6	IGSF6	2.36
Gga.3397.1.S1_at	Gga.42818	N-acetyltransferase 2 (arylamine N-acetyltransferase)	NAT2	2.35
GgaAffx.24574.1.S1_at	Gga.28900	CD274 molecule	CD274	2.34
GgaAffx.24870.1.S1_s_at	Gga.7071	solute carrier family 23 (nucleobase transporters), member 2	SLC23A2	2.34
GgaAffx.22855.1.S1_at	Gga.41632	similar to 4930506M07Rik protein	LOC771423	2.34
GgaAffx.11821.1.S1_s_at	Gga.21297	sterol-C4-methyl oxidase-like	SC4MOL	2.33
Gga.523.1.S1_at	Gga.523	cathepsin L2	CTSL2	2.33
Gga.4285.1.S1_at	Gga.4285	CCAAT/enhancer binding protein (C/EBP), beta	CEBPB	2.33
Gga.483.1.S1_at	Gga.483	JTAP-1	LOC395818	2.32
Gga.9136.1.S1_at	Gga.9136	tumor-associated calcium signal transducer 1	TACSTD1	2.32

Gga.740.1.S1_at	Gga.37849	S100 calcium binding protein A9	S100A9	2.32
Gga.7639.2.S1_a_at	Gga.7639	similar to putative protein product of HMFN0672	LOC422000	2.31
Gga.690.1.S1_at	Gga.690	lymphocyte antigen 86	LY86	2.31
GgaAffx.12417.1.S1_at	Gga.22371	PQ loop repeat containing 2	PQLC2	2.31
GgaAffx.13096.1.S1_at	Gga.18933	Ras association (RalGDS/AF-6) domain family 5	RASSF5	2.31
GgaAffx.12933.1.S1_at	Gga.4188	solute carrier family 25, member 13 (citrin)	SLC25A13	2.3
Gga.16342.1.S1_at	Gga.16342	ribonuclease T2	RNASET2	2.3
GgaAffx.3906.1.S1_s_at	Gga.7601	galactosamine (N-acetyl)-6-sulfate sulfatase (Morquio syndrome, mucopolysaccharidosis type IVA)	GALNS	2.3
Gga.19101.1.S1_at	Gga.19101	Finished cDNA, clone ChEST926n8	---	2.29
GgaAffx.12454.1.S1_s_at	Gga.4593	palmitoyl-protein thioesterase 1 (ceroid-lipofuscinosis, neuronal 1, infantile)	PPT1	2.29
Gga.8822.1.S1_at	Gga.8822	NADH dehydrogenase (ubiquinone) 1 beta subcomplex, 8, 19kDa	NDUFB8	2.28
GgaAffx.3113.1.S1_at	Gga.28865	catechol-O-methyltransferase domain containing 1	COMTD1	2.27
Gga.8003.1.S1_at	Gga.43229	chromosome 20 open reading frame 30	C20orf30	2.27
Gga.9362.1.S1_at	Gga.45795	Transcribed locus	---	2.27
Gga.11477.1.S1_at	Gga.11477	hypothetical LOC418095	LOC418095	2.27
GgaAffx.907.1.S1_at	Gga.14395	NAD kinase	NADK	2.26
Gga.10606.1.S1_at	Gga.10606	suppressor of cytokine signaling 1	SOCS1	2.24
Gga.5666.2.S1_a_at	Gga.46448	Finished cDNA, clone ChEST362o18	---	2.24
GgaAffx.24611.2.S1_s_at	Gga.24333	similar to histamine H3 receptor H3S	LOC428525	2.24
Gga.155.1.S1_s_at	Gga.667	lectin, galactoside-binding, soluble, 3	LGALS3	2.24
Gga.7305.2.S1_a_at	Gga.7305	solute carrier family 31 (copper transporters), member 2	SLC31A2	2.23
GgaAffx.12460.1.S1_s_at	Gga.4677	Niemann-Pick disease, type C2	NPC2	2.23
GgaAffx.9243.1.S1_s_at	Gga.29117	CCR4-NOT transcription complex, subunit 6 /// similar to CCR4-NOT transcription complex, subunit 6	CNOT6	2.22
Gga.3876.1.S1_at	Gga.3876	ATPase, H+ transporting, lysosomal 56/58kDa, V1 subunit B2	ATP6V1B2	2.22
GgaAffx.12510.1.S1_at	Gga.22586	hypothetical protein	LOC770922	2.22

GgaAffx.1121.1.S1_s_at	Gga.22479	UBA domain containing 1	UBAC1	2.21
Gga.12881.1.S1_s_at	Gga.12881	ATPase, class VI, type 11B	ATP11B	2.21
GgaAffx.12149.1.S1_at	Gga.39733	transaldolase 1 /// similar to EPS8-like 2	TALDO1	2.21
GgaAffx.20713.1.S1_s_at	Gga.4704	phosphoribosylglycinamide formyltransferase, phosphoribosylglycinamide synthetase, phosphoribosylaminoimidazole synthetase	GART	2.21
Gga.4139.1.S1_at	Gga.4139	aconitase 1, soluble	ACO1	2.2
Gga.4989.1.S1_s_at	Gga.4989	cytochrome b5 type A (microsomal)	CYB5A	2.2
GgaAffx.9126.5.S1_s_at	Gga.22408	lectin, galactoside-binding, soluble, 8 (galectin 8)	LGALS8	2.2
Gga.13304.1.S1_at	Gga.13304	myelin protein zero-like 3	MPZL3	2.2
Gga.5607.1.S1_at	Gga.5607	Transcribed locus, moderately similar to XP_963881.2 hypothetical protein NCU08122 [Neurospora crassa OR74A]	---	2.2
Gga.17102.1.S1_s_at	Gga.12948	tumor protein D52	TPD52	2.18
Gga.9018.2.S1_at	Gga.43764	similar to KIAA0494 protein	LOC424617	2.18
GgaAffx.11445.1.S1_at	Gga.15890	syntaxin 7	STX7	2.18
GgaAffx.12848.1.S1_at	Gga.30798	leucine-rich PPR-motif containing	LRPPRC	2.18
Gga.3565.1.S1_a_at	Gga.43413	ubiquitin carboxyl-terminal esterase L3 (ubiquitin thiolesterase)	UCHL3	2.16
Gga.4511.1.S1_at	Gga.4511	T-cell, immune regulator 1, ATPase, H+ transporting, lysosomal V0 subunit A3	TCIRG1	2.16
Gga.16491.3.S1_a_at	Gga.16491	MOB1, Mps One Binder kinase activator-like 3 (yeast) /// similar to 2C4D	RCJMB04_1o21	2.16
GgaAffx.3525.1.S1_at	Gga.21980	coagulation factor III (thromboplastin, tissue factor)	F3	2.16
GgaAffx.24771.1.S1_at	Gga.43314	Ras-related GTP binding D	RRAGD	2.15
GgaAffx.21334.1.S1_at	Gga.23699	Finished cDNA, clone ChEST613g9	---	2.15
GgaAffx.5549.1.S1_at	Gga.23512	ATPase, H+ transporting, lysosomal accessory protein 1	ATP6AP1	2.14
GgaAffx.10317.4.S1_s_at	Gga.21681	ATP-binding cassette, sub-family G (WHITE), member 1	ABCG1	2.14
GgaAffx.11995.1.S1_at	Gga.7693	basic leucine zipper and W2 domains 2	BZW2	2.14
Gga.12233.1.S1_at	Gga.12233	N-acylsphingosine amidohydrolase (acid ceramidase)-like	ASAHL	2.14
GgaAffx.7589.1.S1_at	Gga.39420	chemokine (C-X3-C motif)	CX3CR1	2.14

		receptor 1		
Gga.7959.2.S1_a_at	Gga.7959	SH3 domain containing, Ysc84-like 1 (S. cerevisiae)	SH3YL1	2.13
Gga.1114.1.S1_at	Gga.1114	cdk inhibitor CIP1 (p21)	CIP1	2.13
Gga.5128.1.S1_at	Gga.5128	chemokine (C-C motif) ligand 20	CCL20	2.13
GgaAffx.25290.1.S1_s_at	Gga.15725	hypothetical LOC426125	LOC426125	2.12
GgaAffx.6515.1.S1_at	Gga.29911	feline leukemia virus subgroup C cellular receptor family, member 2	FLVCR2	2.12
GgaAffx.5192.1.S1_s_at	Gga.44331	solute carrier family 26, member 5 (prestin)	SLC26A5	2.12
GgaAffx.13196.1.S1_s_at	Gga.43182	gasdermin 1	RCJMB04_34p8	2.11
GgaAffx.2089.1.S1_at	Gga.25854	hypothetical LOC416228	LOC416228	2.11
GgaAffx.6124.1.S1_at	Gga.1326	core 1 synthase, glycoprotein-N-acetylgalactosamine 3-beta-galactosyltransferase, 1	C1GALT1	2.1
Gga.14813.1.S1_at	Gga.14813	Finished cDNA, clone ChEST1014h6	---	2.1
Gga.19791.1.S1_s_at	Gga.24012	low density lipoprotein-related protein 12	LRP12	2.1
Gga.5454.1.S1_at	Gga.5454	abhydrolase domain containing 5	ABHD5	2.09
GgaAffx.12066.1.S1_s_at	Gga.6697	chromatin modifying protein 1B	CHMP1B	2.09
GgaAffx.23085.1.S1_s_at	Gga.35751	RAB3A interacting protein (rabin3)	RAB3IP	2.09
GgaAffx.21211.1.S1_s_at	Gga.8210	vacuolar protein sorting 4 homolog B (S. cerevisiae)	VPS4B	2.08
GgaAffx.11920.1.S1_s_at	Gga.22315	SWI/SNF related, matrix associated, actin dependent regulator of chromatin, subfamily a, member 5	SMARCA5	2.08
GgaAffx.11679.1.S1_s_at	Gga.6959	interleukin 4 receptor	IL4R	2.08
Gga.6387.1.S1_at	Gga.34362	ras-related C3 botulinum toxin substrate 2 (rho family, small GTP binding protein Rac2)	RAC2	2.08
GgaAffx.11711.1.S1_s_at	Gga.7052	synaptosomal-associated protein, 91kDa homolog (mouse)	SNAP91	2.07
GgaAffx.21772.1.S1_s_at	Gga.391	interleukin 10 receptor, beta	IL10RB	2.07
GgaAffx.4296.1.S1_at	Gga.9957	phosphatidylserine decarboxylase	PISD	2.07
GgaAffx.21844.1.S1_s_at	Gga.1917	cadherin 2, type 1, N-cadherin (neuronal)	CDH2	2.07
GgaAffx.11480.1.S1_s_at	Gga.4218	ubiquitin-fold modifier 1	UFM1	2.07
Gga.15124.1.S1_at	Gga.43650	MOB1, Mps One Binder kinase activator-like 1A (yeast)	MOBKL1A	2.06
Gga.9126.2.S1_at	Gga.34665	similar to immunoglobulin-like receptor CHIR-A2 /// similar to	LOC425308	2.06

Gga.9126.2.S1_at	Gga.34665	similar to immunoglobulin-like receptor CHIR-A2 /// similar to immunoglobulin-like receptor CHIR-AB1	LOC425308	2.06
Gga.13662.1.S1_s_at	Gga.17991	programmed cell death 6 interacting protein	PDCD6IP	2.06
GgaAffx.24832.1.S1_s_at	---	---	---	2.05
Gga.2148.4.S1_a_at	Gga.2148	solute carrier family 31 (copper transporters), member 1	SLC31A1	2.05
GgaAffx.12509.1.S1_s_at	Gga.9900	slowmo homolog 2 (Drosophila)	RCJMB04_17b4	2.05
GgaAffx.9520.1.S1_at	Gga.44426	transmembrane protein 128	TMEM128	2.04
Gga.16394.1.S1_at	Gga.47062	complement C4 /// steroid 21-hydroxylase	C4 /// CYP21	2.04
GgaAffx.4536.1.S1_s_at	Gga.30983	Rho GTPase activating protein 12	ARHGAP12	2.04
GgaAffx.11659.1.S1_s_at	Gga.34652	sorcin	RCJMB04_3f15	2.03
Gga.13578.1.S1_at	Gga.38189	Finished cDNA, clone ChEST547d13	---	2.03
Gga.4824.1.S1_at	Gga.4824	ATPase, H+ transporting, lysosomal 13kDa, V1 subunit G1	ATP6V1G1	2.03
GgaAffx.11823.1.S1_s_at	Gga.30127	solute carrier family 7 (cationic amino acid transporter, y+ system), member 6	SLC7A6	2.02
GgaAffx.9018.1.S1_at	Gga.16654	nucleoporin 50kDa	NUP50	2.02
GgaAffx.12319.1.S1_s_at	Gga.16652	hypothetical protein LOC769855	RCJMB04_12m23	2.02
Gga.9377.1.S1_at	Gga.9377	Similar to Expressed sequence AW146242	LOC776358	2.01
GgaAffx.1584.2.S1_s_at	Gga.22709	solute carrier family 9 (sodium/hydrogen exchanger), member 9	RCJMB04_16a12	2.01
Gga.6328.2.S1_s_at	Gga.6328	translocase of inner mitochondrial membrane 17 homolog A (yeast)	TIMM17A	2.01
Gga.1019.1.S1_at	Gga.18911	Finished cDNA, clone ChEST270m22	---	2.01
Gga.2977.1.S1_at	Gga.2977	protein tyrosine phosphatase, receptor-type, Z polypeptide 1	PTPRZ1	2.01
GgaAffx.12005.1.S1_s_at	Gga.4788	similar to Zgc:63829	RCJMB04_8a2	2.01
