# Supplementary material for: Gene signatures in wound tissue as evidenced by molecular profiling in the chick embryo model
Source: BMC Genomics. 2010 Sep 14;11:495. doi: 10.1186/1471-2164-11-495 (PMC2996991; doi:10.1186/1471-2164-11-495)
Supplement: Additional file 3 — (additional Table S3): List of all down-regulated genes (cut off > 0.1). [file 1471-2164-11-495-S3.PDF]

Affymetrix ID	UniGene ID	Gene.Name	Gene Symbol	Fold Change
Gga.9732.1.S1_at	Gga.43260	inter-alpha (globulin) inhibitor H5	ITIH5	-9.09
Gga.3013.1.S1_at	Gga.3013	collagen, type VIII, alpha 1	COL8A1	-6.67
Gga.3652.1.S1_at	Gga.3652	C-type lectin domain family 3, member B	CLEC3B	-4.35
GgaAffx.8087.2.S1_s_at	---	---	---	-4.17
Gga.15966.1.S1_at	Gga.35123	ankyrin 2, neuronal	ANK2	-3.85
Gga.9166.1.S1_at	Gga.9166	Finished cDNA, clone ChEST252j10	---	-3.57
Gga.16835.1.S1_at	Gga.15599	sparc/osteonectin, cwcv and kazal-like domains proteoglycan (testican) 1	SPOCK1	-3.45
Gga.8360.1.S1_at	Gga.36205	Atonal homolog 8 (Drosophila)	ATOH8	-3.45
GgaAffx.26547.1.S1_at	---	---	---	-3.33
Gga.170.1.S1_at	Gga.170	wingless-type MMTV integration site family, member 2B	WNT2B	-3.33
Gga.6141.1.S1_at	Gga.6141	immunoglobin superfamily, member 21	IGSF21	-3.33
Gga.12209.1.S1_at	Gga.12209	Chromosome 4 open reading frame 31	C4orf31	-3.12
GgaAffx.24366.1.S1_at	Gga.23904	laminin, alpha 1	LAMA1	-3.12
Gga.9513.1.S2_at	Gga.9513	Chemokine (C-X-C motif) ligand 12 (stromal cell-derived factor 1)	CXCL12	-3.12
GgaAffx.7505.1.S1_at	Gga.29269	C1q and tumor necrosis factor related protein 1	C1QTNF1	-3.12
Gga.19875.1.S1_at	Gga.23851	plexin A2	PLXNA2	-3.03
Gga.8241.1.S1_at	Gga.8241	transcription factor 21	TCF21	-2.86
Gga.9024.1.S1_at	Gga.43042	chromosome 8 open reading frame 22	C8orf22	-2.86
Gga.618.1.S1_at	Gga.43121	cytochrome P450 1A4	CYP1A4	-2.78
GgaAffx.3294.1.S1_at	Gga.37857	similar to SYT9 protein	LOC423026	-2.7
GgaAffx.4610.1.S1_s_at	Gga.3019	similar to membrane associated guanylate kinase, WW and PDZ domain containing 1	LOC416083	-2.63
Gga.2888.1.S1_at	Gga.2888	fibrillin 1	FBN1	-2.63
GgaAffx.26220.2.S1_s_at	Gga.6559	sema domain, immunoglobulin domain (Ig), short basic domain, secreted, (semaphorin) 3G	SEMA3G	-2.63
Gga.5369.1.S1_at	Gga.39544	sodium channel, nonvoltage-	SCNN1G	-2.56

Gga.5369.1.S1_at	Gga.39544	sodium channel, nonvoltage-gated 1, gamma	SCNN1G	-2.56
Gga.8435.1.S1_at	Gga.8435	Similar to Ecrg4-A protein	LOC771055	-2.56
Gga.18421.1.S1_at	Gga.18421	Finished cDNA, clone ChEST659j16	---	-2.56
GgaAffx.20237.1.S1_s_at	Gga.20551	myc target 1	MYCT1	-2.5
Gga.15323.1.S1_at	Gga.15323	Finished cDNA, clone ChEST408k8	---	-2.33
Gga.1761.1.S1_at	Gga.1761	G protein-coupled receptor 116	GPR116	-2.33
Gga.12140.2.S1_a_at	Gga.12140	EGF-like-domain, multiple 7	EGFL7	-2.27
Gga.2731.1.S1_at	Gga.2731	Finished cDNA, clone ChEST793k15	---	-2.27
Gga.12104.1.S1_at	Gga.12104	receptor (G protein-coupled) activity modifying protein 2	RAMP2	-2.27
Gga.2971.2.S1_s_at	Gga.2971	Glycosyltransferase	AER61	-2.22
Gga.7530.1.S1_at	Gga.7530	Transcribed locus	---	-2.22
GgaAffx.5124.2.S1_s_at	---	---	---	-2.17
GgaAffx.21146.1.S1_at	Gga.44769	Finished cDNA, clone ChEST989g14	---	-2.17
Gga.7994.1.S1_at	---	---	---	-2.13
Gga.12525.1.S1_at	Gga.12525	FRAS1 related extracellular matrix 1	FREM1	-2.13
Gga.14029.1.S1_at	Gga.14029	Solute carrier family 16, member 12 (monocarboxylic acid transporter 12)	SLC16A12	-2.13
Gga.5001.1.S1_at	Gga.5001	cysteine rich transmembrane BMP regulator 1 (chordin-like)	CRIM1	-2.13
GgaAffx.2324.1.S1_at	Gga.47431	chloride channel Kb	CLCNKB	-2.13
Gga.4157.1.S1_at	Gga.4157	EPH receptor A7	EPHA7	-2.08
Gga.6268.1.S1_at	Gga.6268	Finished cDNA, clone ChEST867d11	---	-2.04
Gga.13724.1.S1_at	Gga.13724	Finished cDNA, clone	---	-2
