# Supplementary material for: Gene signatures in wound tissue as evidenced by molecular profiling in the chick embryo model
Source: BMC Genomics. 2010 Sep 14;11:495. doi: 10.1186/1471-2164-11-495 (PMC2996991; doi:10.1186/1471-2164-11-495)

A, Network 5 (upregulated genes)

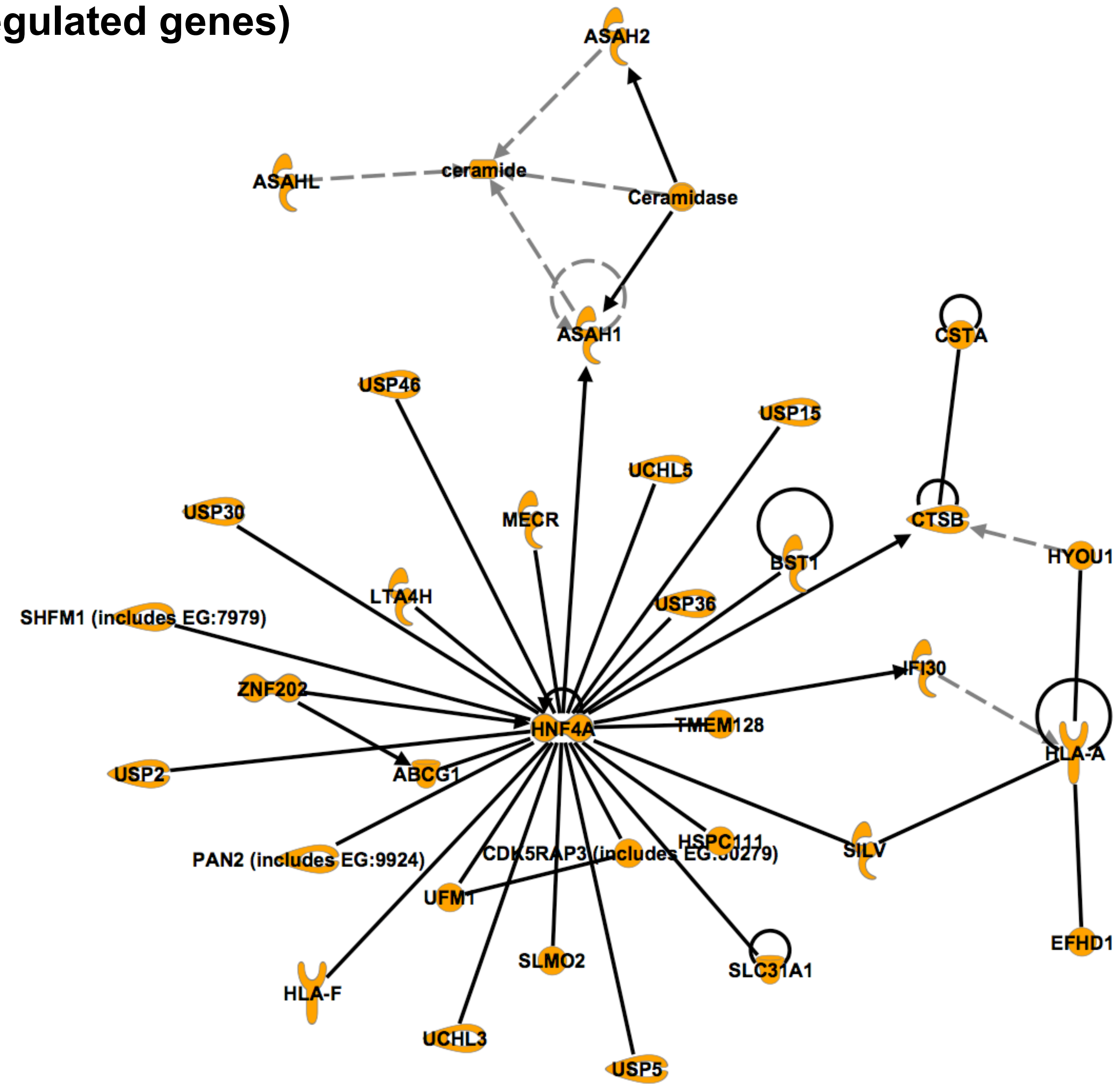

B, Network 6 (upregulated genes)

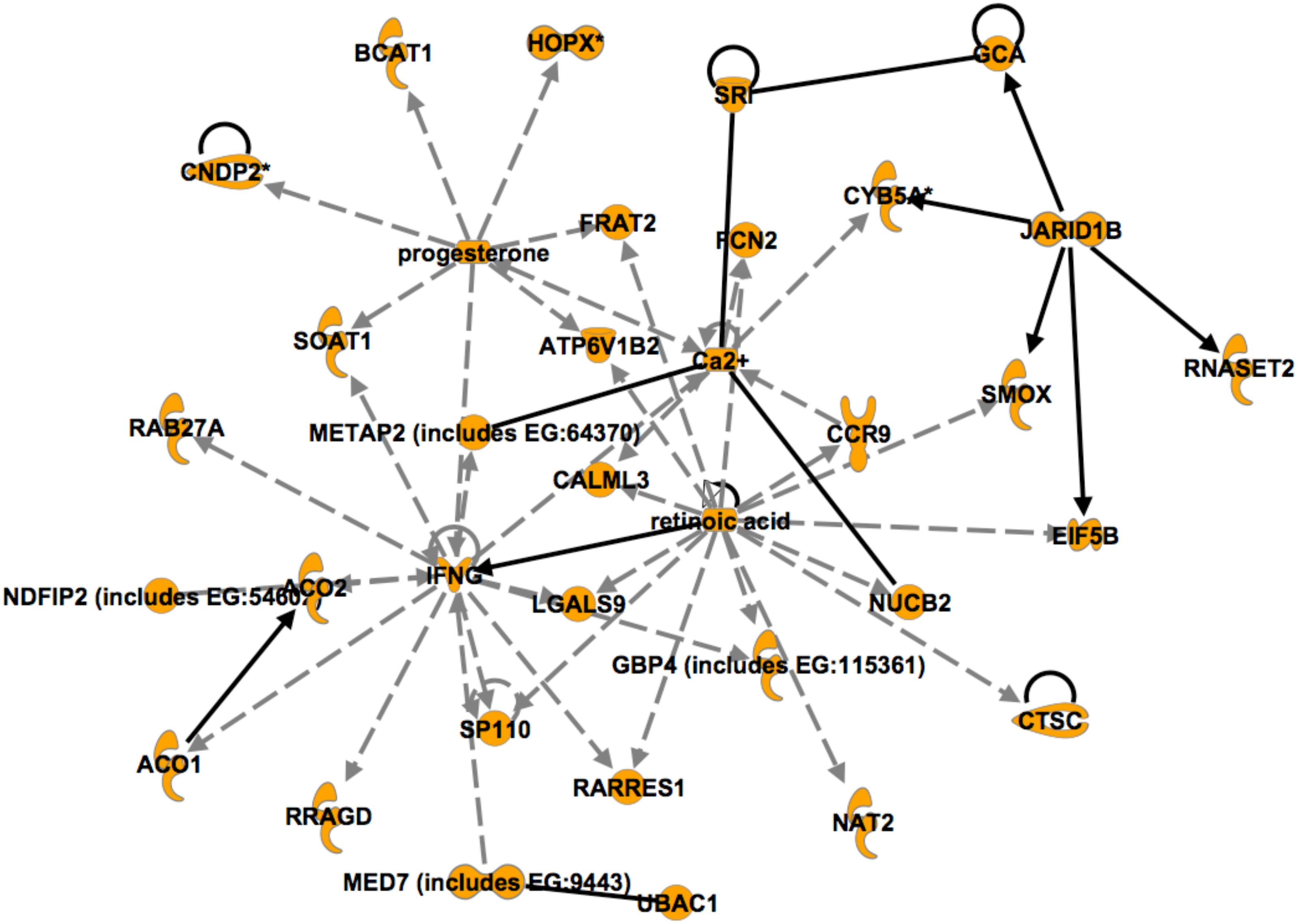

C, Network 7 (upregulated genes)

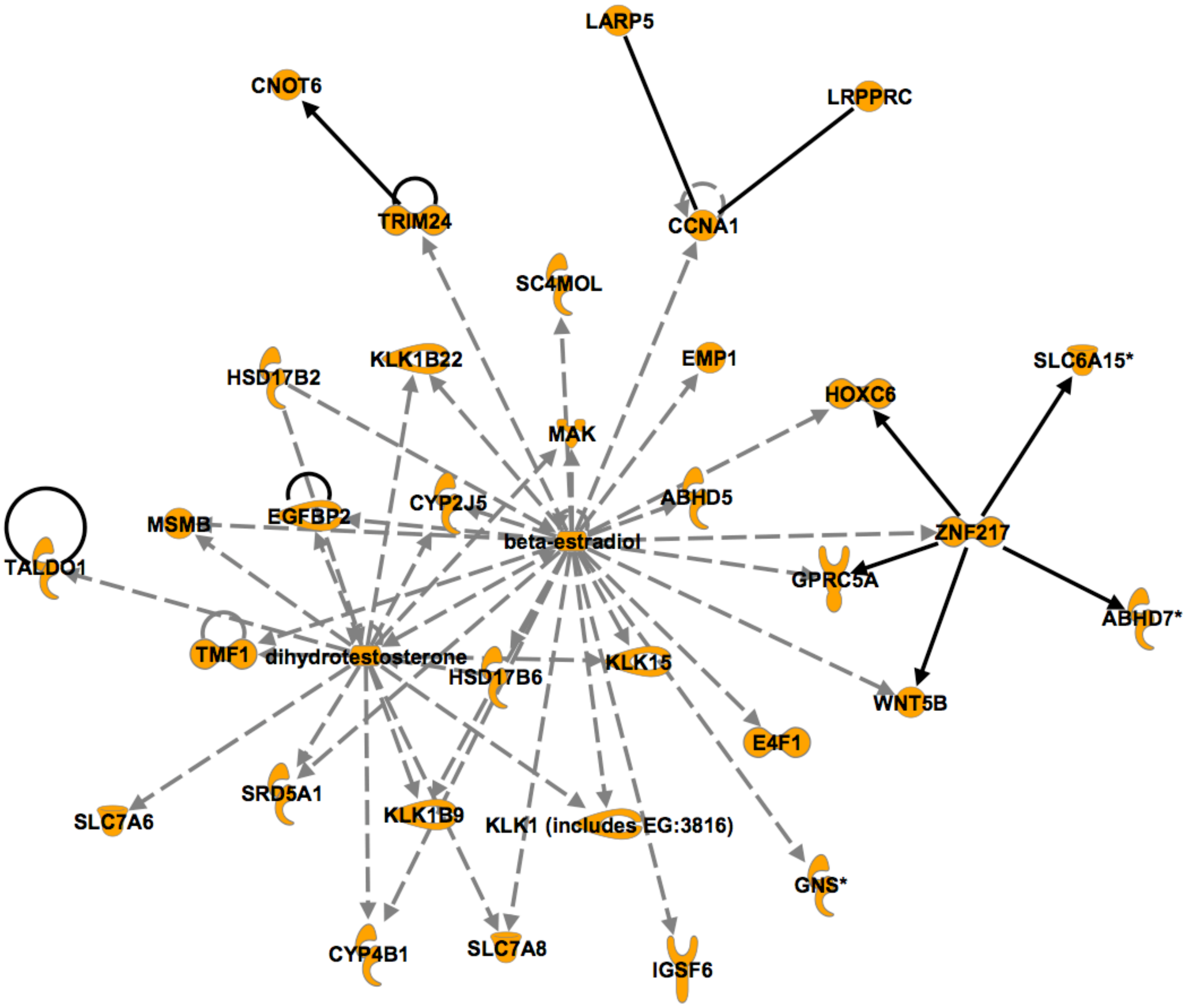

D, Network 8 (upregulated genes)

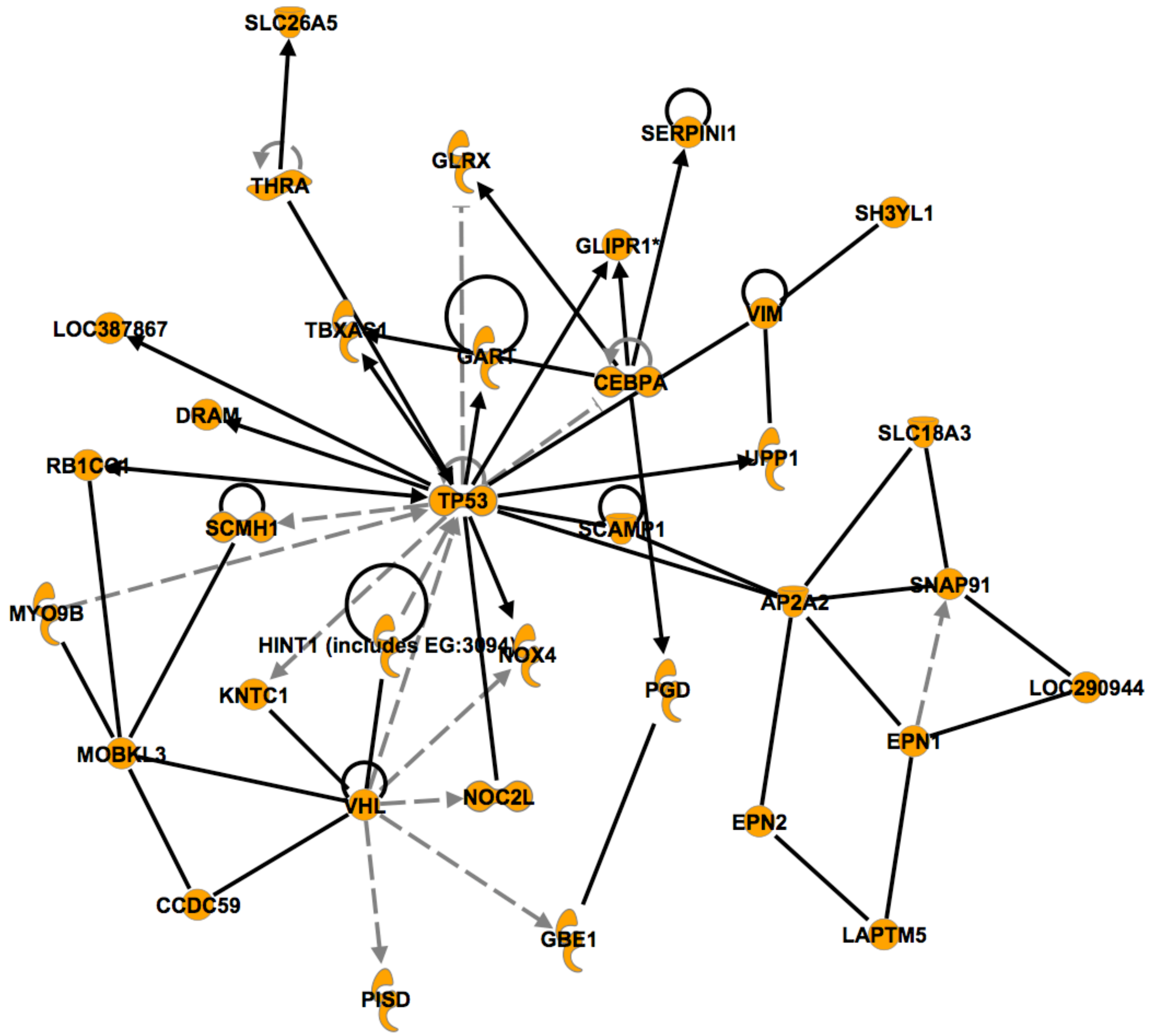

E, Network 9 (upregulated genes)

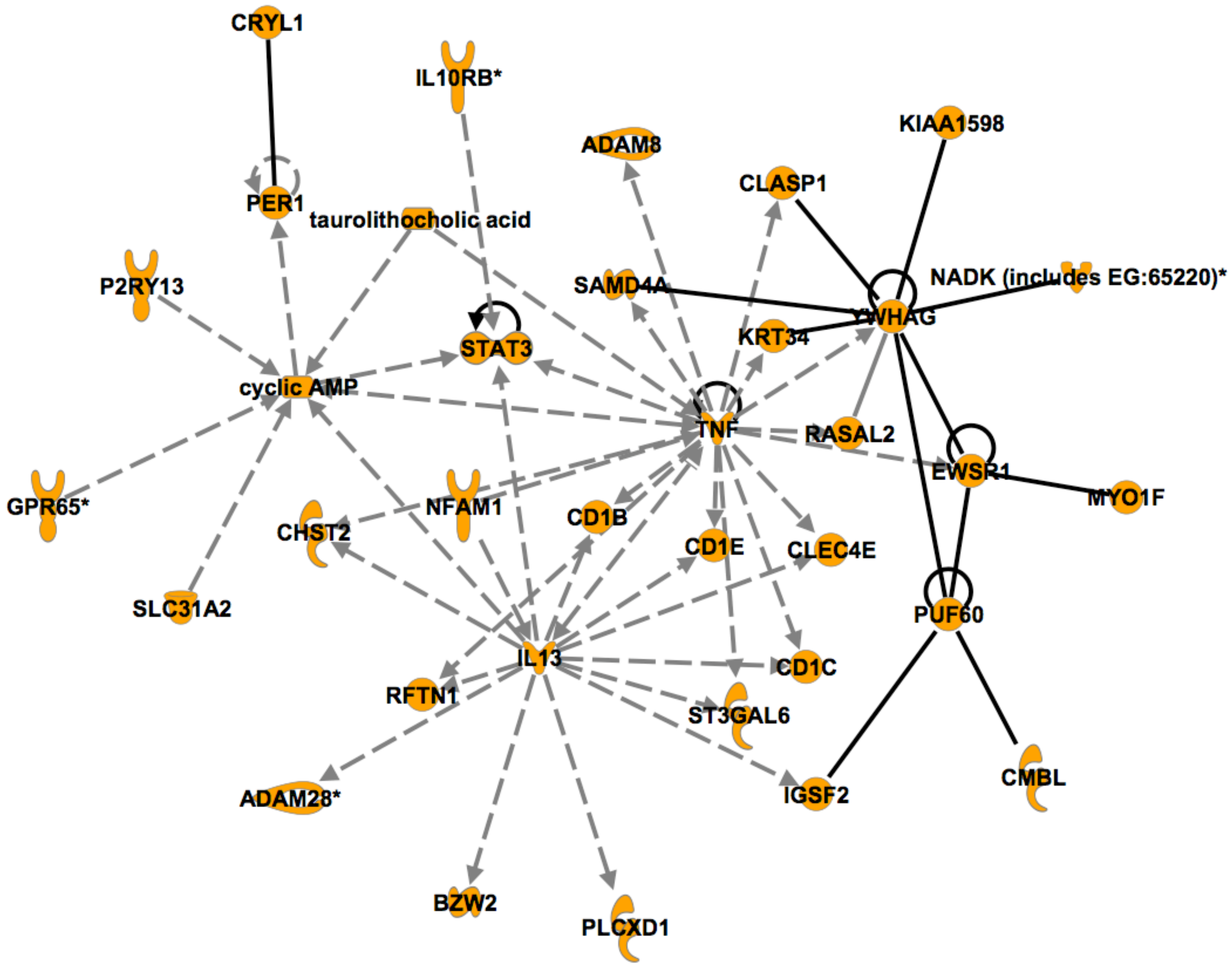

F, Network 10 (upregulated genes)

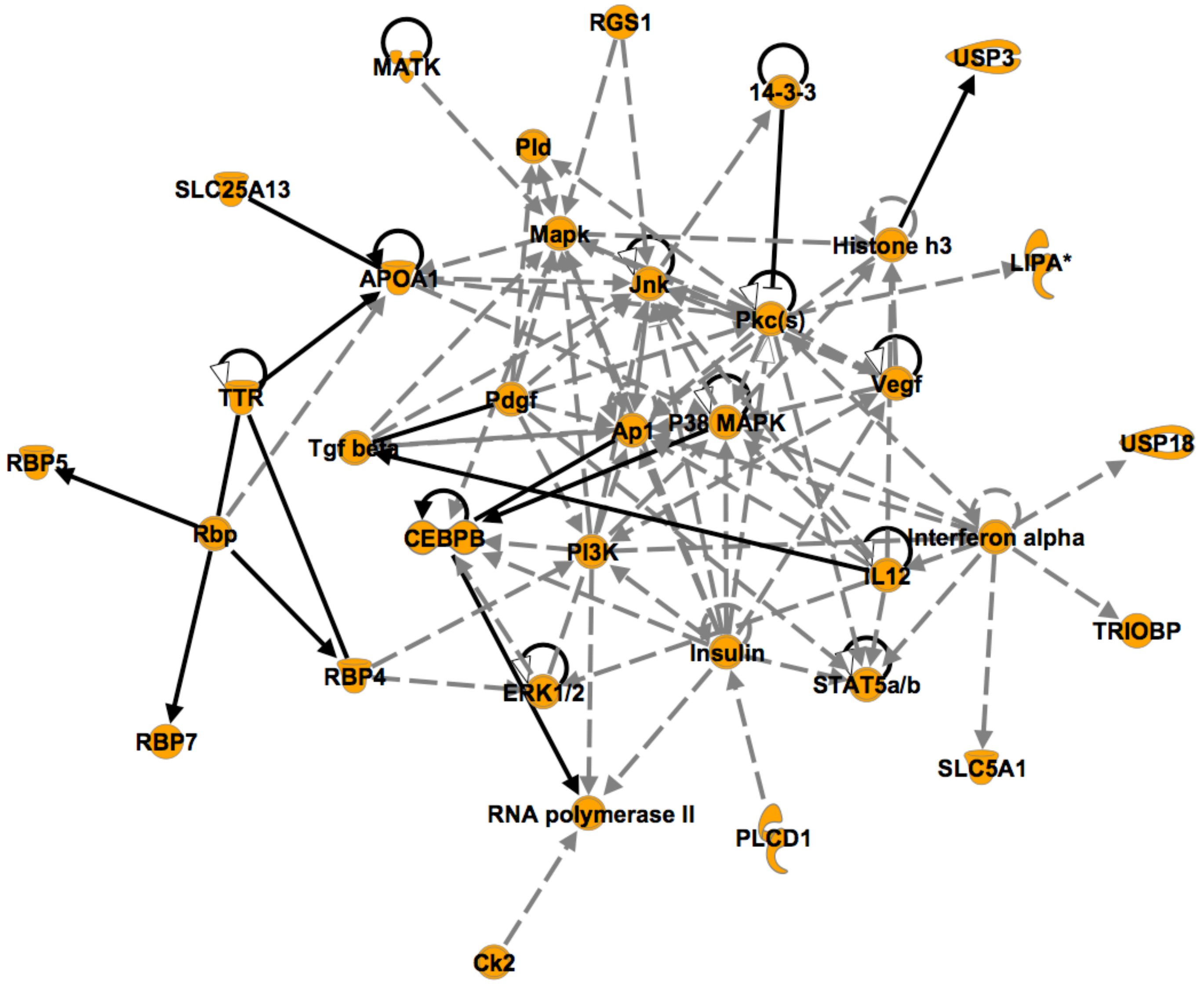

G, Network 11 (upregulated genes)

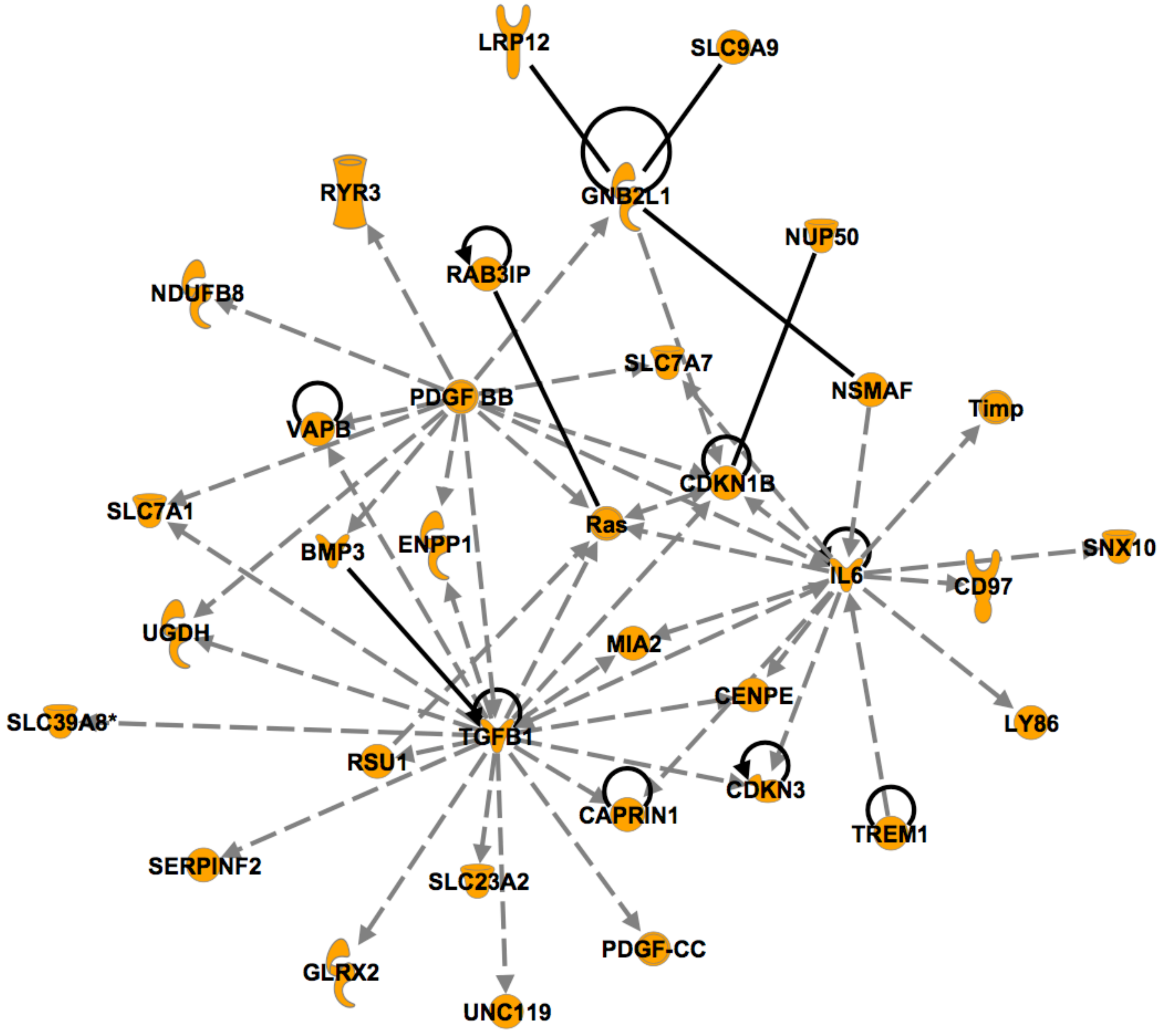

H, Network 12 (upregulated genes)

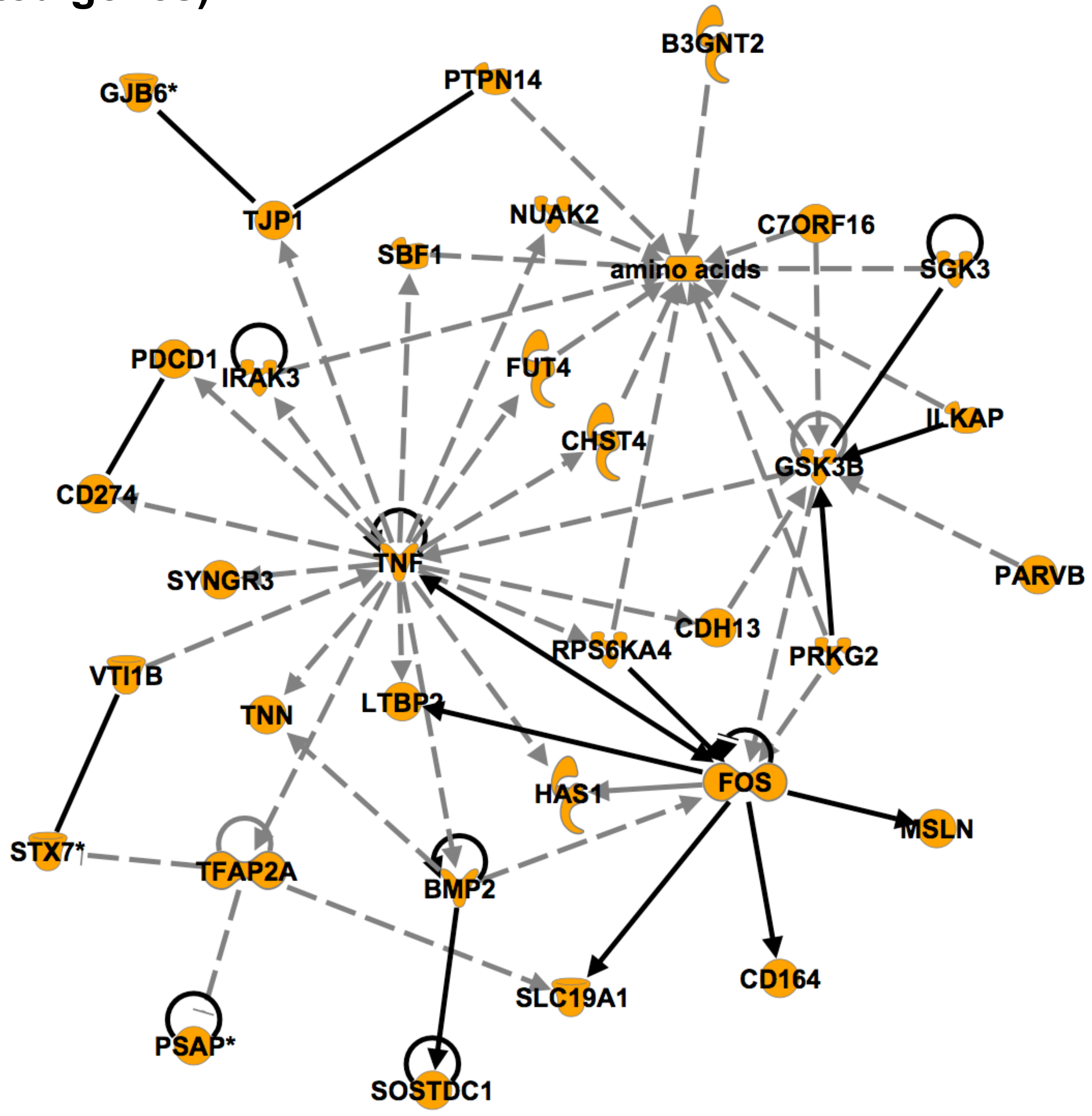

I, Network 1 (downregulated genes)

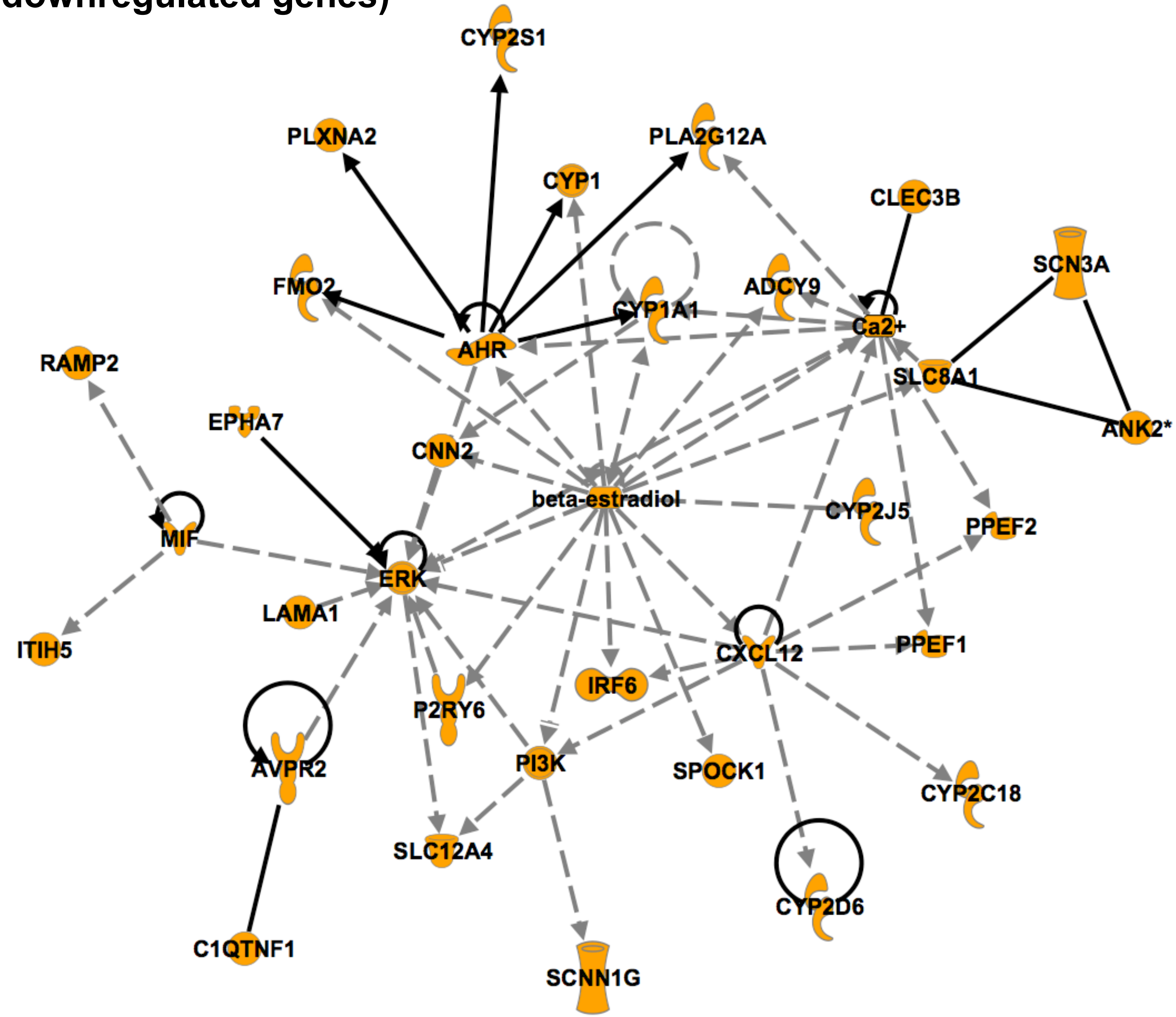

J, Network 2 (downregulated genes)

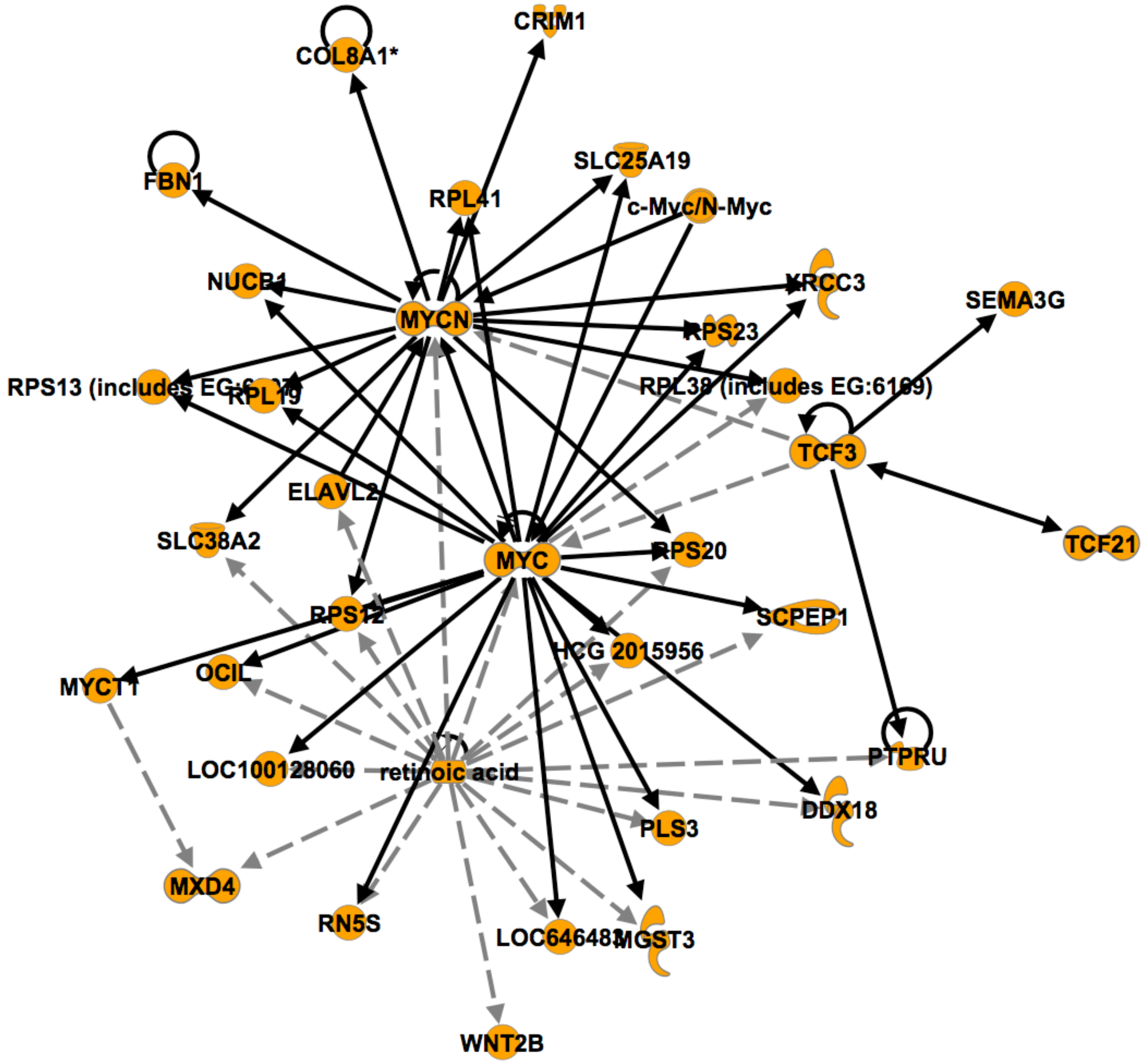

Supplement: Additional file 5 — (additional Figure S1A-J): Representation of networks from the IPA for up-(A-H) or down-regulated genes (I-J). [file 1471-2164-11-495-S5.PDF]
